# Genome-wide in silico characterization and stress induced expression analysis of BcL-2 associated athanogene (*BAG*) family in *Musa* spp.

**DOI:** 10.1038/s41598-021-04707-5

**Published:** 2022-01-12

**Authors:** Ashutosh Dash, Siddhesh B. Ghag

**Affiliations:** School of Biological Sciences, UM-DAE Centre for Excellence in Basic Sciences, University of Mumbai Campus, Kalina, Santacruz (East), Mumbai, 400 098 India

**Keywords:** Plant sciences, Systems biology

## Abstract

Programmed cell death (PCD) is a genetically controlled process for the selective removal of damaged cells. Though understanding about plant PCD has improved over years, the mechanisms are yet to be fully deciphered. Among the several molecular players of PCD in plants, B cell lymphoma 2 (Bcl-2)-associated athanogene (BAG) family of co-chaperones are evolutionary conserved and regulate cell death, growth and development. In this study, we performed a genome-wide in silico analysis of the *MusaBAG* gene family in a globally important fruit crop banana. Thirteen *MusaBAG* genes were identified, out of which *MusaBAG1*, *7* and *8* genes were found to have multiple copies. *MusaBAG* genes were distributed on seven out of 11 chromosomes in banana. Except for one paralog of *MusaBAG8* all the other 12 proteins have characteristic BAG domain. MusaBAG1, 2 and 4 have an additional ubiquitin-like domain whereas MusaBAG5-8 have a calmodulin binding motif. Most of the MusaBAG proteins were predicted to be localized in the nucleus and mitochondria or chloroplast. The in silico* cis*-regulatory element analysis suggested regulation associated with photoperiodic control, abiotic and biotic stress. The phylogenetic analysis revealed 2 major clusters. Digital gene expression analysis and quantitative real-time RT-PCR depicted the differential expression pattern of *MusaBAG* genes under abiotic and biotic stress conditions. Further studies are warranted to uncover the role of each of these proteins in growth, PCD and stress responses so as to explore them as candidate genes for engineering transgenic banana plants with improved agronomic traits.

## Introduction

Programmed cell death (PCD) is a genetically controlled process for the selective removal of damaged and unwanted cells. Like in animals, PCD plays an important role in plant development and response to both abiotic and biotic stress. Though there exists similar molecular and biochemical hallmarks of PCD in plants to that in animals, some key steps (phagocytosis) and important regulators (caspase) were absent in plants. B cell lymphoma 2 (Bcl-2) family of proteins in animals play a crucial role in deciding the fate of cells by regulating the proapoptotic and antiapoptotic signals^1^. However, Bcl-2 proteins are absent in plants. In 1995, another protein called Bcl-2-associated athanogene (BAG1) was identified in animals and found to trigger the antiapoptotic function of Bcl-2 proteins^2^. Homologs of BAG proteins are present in plants and are known to be critically regulating cell death processes^3^.

BAG family of proteins are co-chaperones functioning as molecular switches, regulating the function of molecular chaperones, mainly HSP70/HSC70^4^. These proteins are involved in several cellular pathways ranging from PCD to tumorigenesis^5^. All BAG proteins consist of a characteristic conserved BAG domain (BD) near the C-terminal region. BD interacts with the ATPase domain of the HSP70/HSC70 molecular chaperones^4^. The *Arabidopsis* genome encodes for eight BAG proteins (AtBAG 1–8). Three out of the seven BAGs identified in *Arabidopsis* contain a calmodulin binding motif and the rest four contain an ubiquitin-like (UBL) domain in addition to the BD^6^. Expressed sequence tags (EST) database of *Arabidopsis*, displayed the highest expression level of *AtBAG3* followed by *AtBAG1, 4* and *7* whereas *AtBAG2, 5, 6* and *8* have the least expression level^6^. Transcript levels of *AtBAG4* and *6* were observed to be much higher in young and actively growing tissues, other than the leaves indicating its role in the developmental process^3,6^. *AtBAG4* is known to confer abiotic cold-stress tolerance whereas *AtBAG6* is involved in heat-stress and also inducing PCD^3,7^. Plant *BAG* genes are regulated under biotic stress and upon pathogenic challenge. *BAG4* was induced in the leaf tissue of *Medicago truncatula* when infected with bacterial pathogen *Xylella fastidiosa*^3^. Similarly, in the seedlings of *Hordeum vulgare*, *BAG1* was induced when challenged with *Blumeria*^3^. The transcript levels of *AtBAG6* were elevated on treatment with salicylic acid in *Arabidopsis*. AtBAG6 is proteolytically cleaved by aspartyl proteases to trigger autophagy and induce PCD, conferring fungal disease resistance^8^. AtBAG7 is an endoplasmic reticulum (ER) localized protein, playing a role in unfolded protein response (UPR) signal transduction. Under normal condition it remains associated with the luminal binding protein (AtBiP) and during ER stress condition it dissociates from AtBiP and gets translocated to the nucleus to interact with WRKY-DNA binding protein [WRKY29]^9^. AtBAG5 is a mitochondrial localized protein which when present in abundance induces reactive oxygen species (ROS) production. AtBAG5 sequesters HSC70 protein and therefore the low levels of free HSC70 is involved in ROS alleviation and PCD. AtBAG5 mutants showed delayed leaf senescence^10^. AtBAG1 levels are self-regulated and its levels are important for normal growth and development of the plant. It has been shown to be involved in proteasomal degradation of misfolded and unimported plastid proteins assisted by HSC70-4^11^. A BAG domain containing protein HSG1 in grapevine was induced at 45 °C for 60 min in both leaves and berries. Moreover, when HSG1 was overexpressed in *Arabidopsis* faster floral transition and triggering of flowering promoter CONSTANS was observed without any morphological changes than the control plant indicating its role in photoperiodism^12^. In a previous study, overexpressing native ubiquitin-like domain-containing protein *BAG1* coding sequence in banana plants conferred resistance to Fusarium wilt disease^13^.

In this work, we present the first comprehensive in silico analysis of the *BAG* gene family from banana (*Musa* spp. AA genome) and experimentally shown their differential expression levels under biotic and abiotic stress conditions. To gain more insight, we studied the gene structure, organization, protein structural properties, phylogeny, and analyzed the expression profiles of the *BAG* gene family in banana plants under stress conditions. This conscientious analysis of *BAG* genes in banana plants provides a strong ground for further functional characterization of each of these genes so as to utilize them for developing elite varieties resilient to abiotic and biotic stress.

## Results

### Musa database search for BAG domain containing proteins

**Figure 1:**
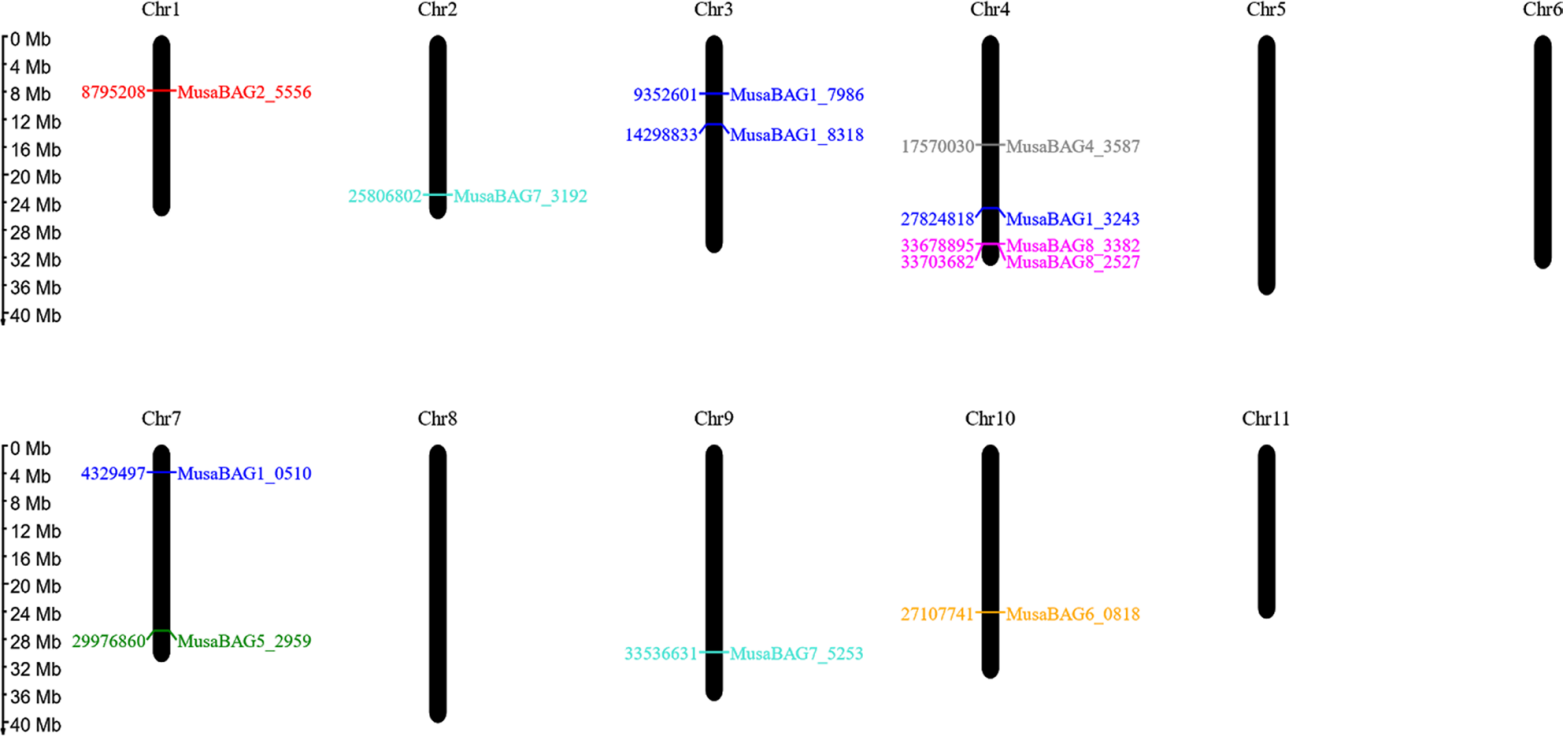
Chromosomal map showing the distribution of the *MusaBAG* gene family members on the A genome of banana (*Musa acuminata* DH-Pahang AA type) using Map2GeneChrom v2.1 tool (http://mg2c.iask.in).

The *Arabidopsis BAG* genes (*AtBAG1-AtBAG8*) were used as query sequences to search for its homologs in the banana genome database. A total of 13 *BAG* genes were identified in the banana genome which were further categorized into 7 different types (Table 1) based on the *AtBAG* sequences. There were no homologs of *AtBAG3* found in the banana genome. Most of the *MusaBAG* genes are present on the chromosome number 3, 4, and 7. But some *BAG* genes were also identified on the chromosomes 1, 2, 9 and 10 (Fig. 1). Out of the 13 *BAG* genes *MusaBAG1, 7* and *8* genes were found to have multiple copies in the genome of banana. *MusaBAG1* has four different copies (paralogous genes) present on three different chromosomes (chr. 3, 4 and 7). Similarly, *MusaBAG7* gene has two paralogs each on chromosome 2 and 9. *MusaBAG8*, exists as two different mRNA transcript variants located on the locus LOC103982527 of chromosome 4. A truncated paralogous gene, also denoted as *MusaBAG8* (based on similarity search using *AtBAG8*), was also found at locus LOC103983382 of the same chromosome. The *MusaBAG* genes encode for a 200–450 amino acid residue protein, except *MusaBAG6*, which encodes for an 1119 amino acid residue protein. Some of the characteristics of *MusaBAGs*, such as length of protein and localization, are similar to BAG genes of *Arabidopsis* and rice, except for the copy numbers of the gene in the respective genomes. Most of the MusaBAG proteins have an isoelectric point (pI) in the alkaline pH range i.e., between pH of 9–10, whereas MusaBAG5 and 6 have a pI in the acidic range and MusaBAG4 and 8 in neutral.

**Table 1:** Annotation, accessions, amino acids, molecular weight and pI of *MusaBAG* genes.

Name	Locus	Nucleotide accession number	Protein accession number	Amino acids	Molecular weight (kDa)	Isoelectric point (pI)
MusaBAG1	LOC103983243	XM_009400456.2	XP_009398731.1	340	38.42	9.64
MusaBAG1	LOC103990510	XM_009409679.2	XP_009407954.1	327	36.55	9.55
MusaBAG1	LOC103977986	XM_009393675.2	XP_009391950.1	319	35.57	9.65
MusaBAG1	LOC103978318	XM_009394078.2	XP_009392353.1	318	37.13	9.48
MusaBAG2	LOC103995556	XM_018825077.1	XP_018680622.1	337	37.16	9.25
MusaBAG4	LOC103973587	XM_009388204.2	XP_009386479.2	238	26.24	6.48
MusaBAG5	LOC103992959	XM_009412890.2	XP_009411165.1	221	24.41	4.67
MusaBAG6	LOC104000818	XM_009422955.2	XP_009421230.1	1119	125.5	5.06
MusaBAG7	LOC103975253	XM_009390167.2	XP_009388442.1	433	48.42	8.98
MusaBAG7	LOC103973192	XM_009387694.2	XP_009385969.1	429	49.01	9.34
MusaBAG8	LOC103982527-X1	XM_009399476.2	XP_009397751.1	401	44.99	7.12
MusaBAG8	LOC103982527-X2	XM_018825107.1	XP_018680652.1	364	40.93	7.03
MusaBAG8	LOC103983382	XM_009400601.2	XP_009398876.2	191	21.63	9.19

### Conserved amino acid sequence analysis

**Figure 2:**
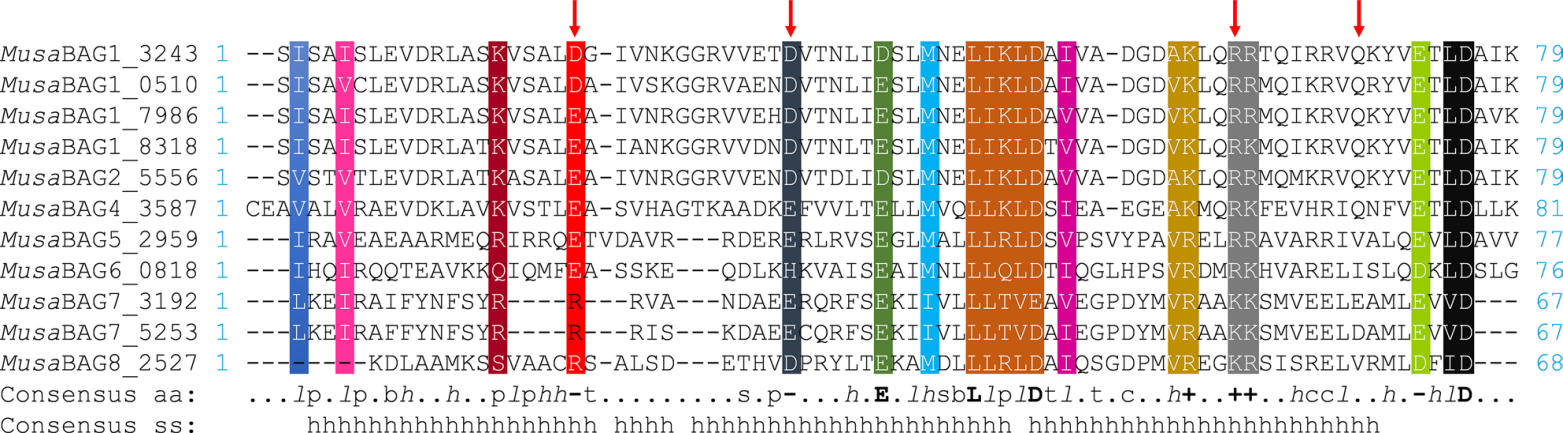
Multiple sequence alignment of BAG domain of MusaBAG proteins depicting conserved amino acid residues. The amino acids glutamate/aspartate (D/E), arginine (R) and glutamine (Q) (depicted as red downward arrow) are the residues possibly interacting with the ATPase domain of Hsc70 similar to the mammalian counterpart BAG1. Other characteristically conserved residues across all MusaBAGs are highlighted in grey.

**Figure 3:**
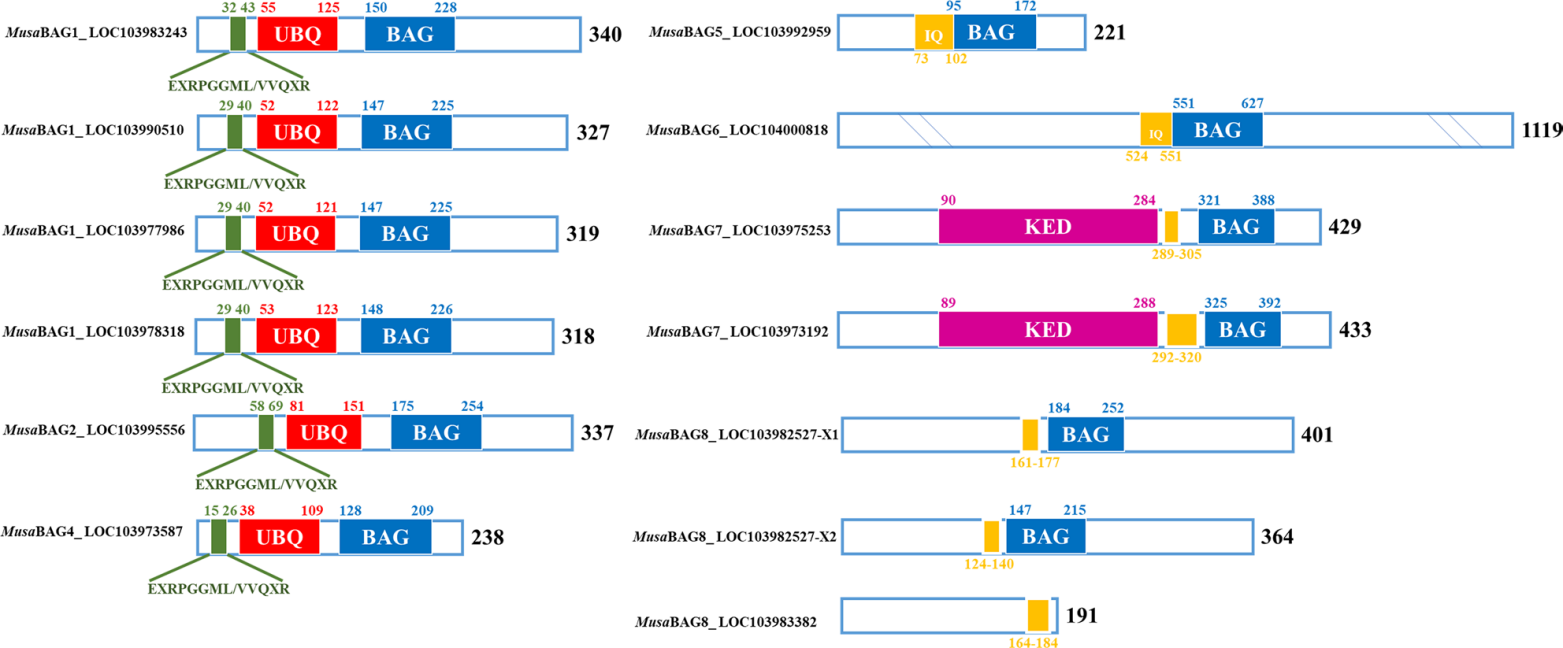
Identification of the conserved domains and motifs present in MusaBAG proteins. All the MusaBAG proteins except MusaBAG8 (LOC103983382) showed the typical BAG domain (depicted in blue). The ubiquitin-like domain (UBQ, depicted in red) was present in MusaBAG1, 2 and 4 whereas the calmodulin-binding motif (IQ, depicted in yellow) was present in MusaBAG5, 6, 7 and 8. KED sequence motif (depicted in magenta) was present in MusaBAG7. A conserved sequence motif of 12 amino acids was present in MusaBAG1, 2 and 4 (depicted in green).

Plant *BAG* genes have a characteristic BAG domain (BD) that is typically 70–80 amino acid residues long. A multiple sequence alignment of the BAG domain of the *MusaBAG* genes showed conserved residues, and probably are vital for their interaction with the chaperones (Fig. 2). Amongst the three helices of the BAG domain, amino acid residues were highly conserved in the second and third helix. In this region, individual residues as well as stretch of residues were conserved in all the BAG domains of MusaBAG proteins. Most of the conserved residues observed here are either hydrophobic residues like valine, isoleucine, leucine, or charged amino acid residues like glutamate, aspartate, arginine and lysine. The LIKLD stretch was conserved in MusaBAG1 paralogs and MusaBAG2 whereas MusaBAG4 have LLKLD. MusaBAG5, 6 and 8 have LLR/QLD as a conserved motif and MusaBAG7 have LLTVE/D. MusaBAG1, 2, and 4 are very similar with respect to their domain conservation and organization. Towards their N-terminus, there is a characteristic conserved sequence motif of 12 amino acids, ExRPGG[ML/VV]QxR (Fig. 3). Although the function of this motif is unknown, this characteristic sequence motif is also present in homologous BAG-domain containing proteins of *Arabidopsis*, rice and *Medicago truncatula*^6^. Apart from the conserved sequence motif and BAG domain, MusaBAG1, 2, and 4 have a conserved ubiquitin-like domain (UBQ). MusaBAG5-8 has a conserved calcium-free calmodulin binding motif, called the IQ-motif, present very close to the BAG domain (Fig. 3). However, *MusaBAG8* (LOC103983382) did not have the characteristic BAG domain but had an IQ-calmodulin binding motif. Both the paralogs of MusaBAG7, have a conserved KED sequence motif. This sequence motif consists of repeated stretches of highly charged amino acids like lysine (K), glutamate (E) and aspartate (D) (Fig. 3).

### Structural analysis

**Figure 4:**
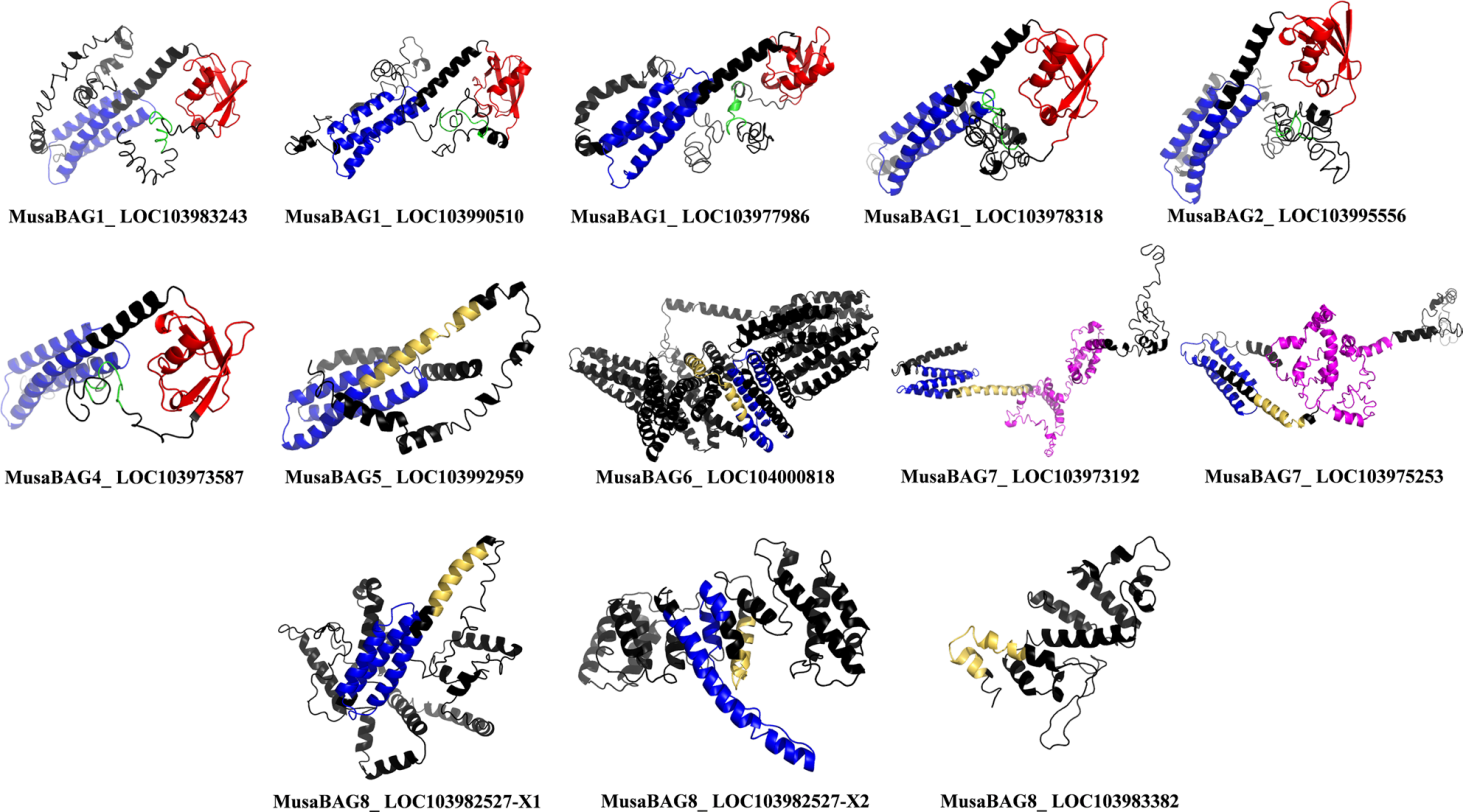
Prediction of three-dimensional structure of MusaBAG proteins. The structures were predicted using the I-TASSER online tool and the ones with the best C-score were represented in the form of ribbon-model. The BAG domains of all the MusaBAG proteins showed characteristic three alpha helix bundle (depicted in blue). The ubiquitin-like domain (UBQ, depicted in red), the calmodulin-binding motif (IQ, depicted in yellow) and KED sequence motif (depicted in magenta) were observed. A conserved sequence motif of 12 amino acids (ExRPGG[ML/VV]QxR) was depicted in green.

The structural analysis of the *Musa*BAG proteins was done based on the predicted structures, obtained by submitting individual protein sequences to I-TASSER server, for template based iterative simulation for prediction of structures. The predicted structures with highest C-score, from the information given by the server, were selected as representative structures for further analysis (Fig. 4). The structures of MusaBAG1 (all the four paralogs), MusaBAG2 and MusaBAG4 were comparable, with respect to the presence and organization of the major domains. These structures have a highly organized BAG domain and ubiquitin-like domain. The N-terminal characteristic 12 amino acid conserved sequence motif present in MusaBAG1, MusaBAG2 and MusaBAG4 (as described in the previous section) was found to be a hairpin loop. The ubiquitin-like domain showed high conservation, with strictly four β-sheets and two α-helices. Amongst the four β-sheets, two were organized as central β-sheets, with one α-helix on each side of the β-sheet. This type of ubiquitin-like domain structure was very similar to the ubiquitin-like domain of yeast ribosomal biogenesis protein Ytm1^14^. The BAG domain of all the MusaBAG proteins consists of the typical three α-helix bundle structure, similar to as described by Sondermann et al.^15^. The IQ-calmodulin binding motif consists of two small α-helices connected by a hairpin loop, which is in continuation with the first helix of the BAG domain, as observed in MusaBAG5, 6, and 8. In MusaBAG7, this motif was a complete single α-helix, which is continued as the first helix of the BAG domain. The KED sequence motif, which is present in both the paralogs of MusaBAG7, was structurally consisting of multiple α-helices connected by multiple hairpin loops. Structurally, this sequence motif showed interaction pockets similar to EF-hand like proteins, which upon docking showed probable calcium and magnesium binding sites (Supplementary Information S1).

### Phylogenetic analysis and classification

**Figure 5:**
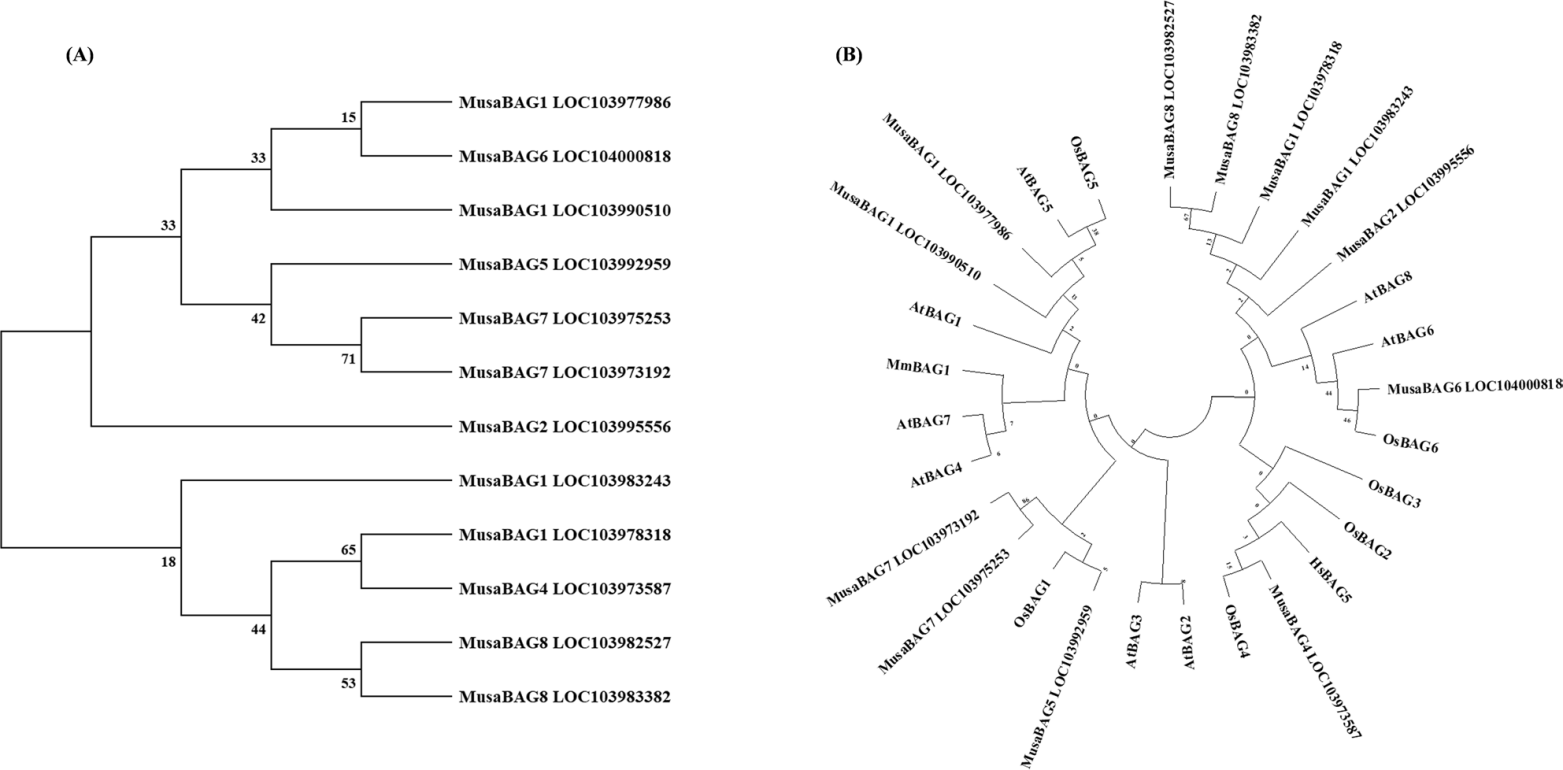
Phylogenetic analysis of the MusaBAG proteins. (**A**) The neighbor joining phylogenetic tree of the full length MusaBAG protein sequence (MusaBAG1_ LOC103983243: XM_009400456.2, MusaBAG1_LOC103990510: XM_009409679.2, MusaBAG1_LOC103977986: XM_009393675.2, MusaBAG1_LOC103978318: XM_009394078.2, MusaBAG2_LOC103995556: XM_018825077.1; MusaBAG4_LOC103973587: XM_009388204.2, MusaBAG5_LOC103992959: XM_009412890.2, MusaBAG6_LOC104000818: XM_009422955.2, MusaBAG7_LOC103975253: XM_009390167.2, MusaBAG7_LOC103973192: XM_009387694.2, MusaBAG8_LOC103982527: XM_009399476.2; MusaBAG8_LOC103983382: XM_009400601.2) was constructed using MEGA version X, with 1000 bootstrap replications. The numbers marked near each branch indicates the percentage of replicate trees in which the associated taxa clustered together in the bootstrap test. (**B**) The neighbor joining phylogenetic tree constructed by taking BAG proteins of *Arabidopsis* (AtBAG1: NP_200019.2, AtBAG2: BAB10172.1, AtBAG3: NP_196339.1, AtBAG4: NP_190746.2, AtBAG5: NP_172670.2, AtBAG6: NP_182147.1, AtBAG7: NP_201045.1, AtBAG8: NP_189577.2), rice (OsBAG1: XP_015651322.1, OsBAG2: XP_015650279.1, OsBAG3: XP_015641134.1, OsBAG4: XP_015621585.1, OsBAG5: XP_015624985.1, OsBAG6: XP_015622983.1), banana (protein sequences same as in **A**), mouse BAG1 (MmBAG1: Q60739.3) and human BAG5 (HsBAG5: NP_001015048.1). The tree was constructed using MEGA version X, with 1000 bootstrap replications. The numbers marked near each branch indicates the percentage of replicate trees in which the associated taxa clustered together in the bootstrap test.

Using the neighbor-joining method, phylogenetic analysis of all BAG proteins from banana (MusaBAG) was performed. From this phylogenetic tree, it was observed that there are two major phylogenetic clades of MusaBAG proteins (Fig. 5A). In both the clades MusaBAG1 was present, suggesting that MusaBAG1 originated or derived from the last common ancestral protein. MusaBAG1_LOC103983243 and MusaBAG1_LOC103978318 were clustered in one clade whereas MusaBAG1_LOC103990510 and MusaBAG1_LOC103977986 were in the other clade. MusaBAG4 and MusaBAG1_LOC103978318 were grouped as one terminal monophyletic clade whereas both the MusaBAG8 were grouped as another monophyletic clade. Similarly, both the MusaBAG7 were grouped as one monophyletic clade, which is in turn branched with MusaBAG5. MusaBAG6 and MusaBAG1_LOC103990510 were grouped as one monophyletic clade. Interestingly, MusaBAG2, which is structurally similar to MusaBAG1 and MusaBAG4 was an out-group to MusaBAG1 (LOC103990510 and LOC103977986), MusaBAG6 and MusaBAG7.

Another un-rooted circular phylogenetic tree was constructed using neighbor-joining method of all BAG proteins from banana (MusaBAG), *Arabidopsis* (AtBAG) and rice (OsBAG) along with a BAG1 from mouse (MmBAG1) and BAG5 from human (HsBAG5) (Fig. 5B). Generally, BAG1, 5 and 7 were clustered in one clade whereas BAG4, 6, 8 were clustered in another clade. However, 2 out of the 4 copies of MusaBAG1 proteins were clustered along with MusaBAG8 proteins. HsBAG5 clustered in a distant clade than plant BAG5 proteins. An unusual observation from this phylogenetic tree was that BAG2, and 3 from rice and banana are clustered as closely related proteins whereas their homologs from *Arabidopsis* were clustered in a separate clade. In this phylogenetic tree MusaBAG2 was clustered along with MusaBAG8, AtBAG8 and MusaBAG1 (LOC103983243 and LOC103978318), which was different compared with the phylogenetic analysis done only for MusaBAG sequences. Thus it was difficult to trace back the origin and evolutionary trajectory of MusaBAG2 based on this phylogenetic analysis.

### Subcellular localization

Subcellular localization of proteins is important to fairly ascertain their function. Several online tools are available to predict protein localization in the cell; that utilizes protein characteristics such as amino acid composition, signal peptides, homology based prediction and combination of these. The subcellular localization of MusaBAG proteins were predicted using DeepLoc 1.0, PSORT, Yloc, Target P, WoLF PSORT and Plant PLoc servers. According to these predictions all the MusaBAG proteins were destined to the nucleus (Table 2). MusaBAG1 proteins were predicted to be present both in nucleus and mitochondria. MusaBAG5, MusaBAG7 (GenBank accession ID—XM_009390167.2) and MusaBAG8 were also shown to be localized in the chloroplast, mitochondria or microbody (peroxisomes). MusaBAG6 was exclusively predicted to be localized in the nucleus by all the 6 servers. MusaBAG4 and MusaBAG7 (GenBank accession ID—XM_009387694.2) were predicted to be localized in the cytoplasm as well.

**Table 2:** Prediction of the subcellular localization MusaBAG proteins (confidence scores mentioned in parenthesis).

Name	Accession number	DeepLoc 1.0	PSORT	Yloc	Target P	WoLF PSORT	Plant PLoc
MusaBAG1	XM_009400456.2	Nucleus (0.818)	Mitochondrial matrix space (0.450)	Nucleus (0.63)	Other (0.995)	Mitochondria/nucleus	Nucleus
MusaBAG1	XM_009409679.2	Nucleus (0.6753)	Mitochondrial matrix space (0.437)	Nucleus (0.63)	Other (0.9879)	Mitochondria	Nucleus
MusaBAG1	XM_009393675.2	Nucleus (0.61)	Cytoplasm (0.650)	Nucleus (0.24)	Other (0.9936)	Mitochondria	Nucleus
MusaBAG1	XM_009394078.2	Nucleus (0.6638)	Mitochondrial matrix space (0.480)	Nucleus (0.58)	Other (0.9834)	Mitochondria/chloroplast	Nucleus
MusaBAG2	XM_018825077.1	Nucleus (0.6141)	Mitochondrial matrix space (0.450)	Chloroplast (0.36)	Other (0.9632)	Nucleus	Nucleus
MusaBAG4	XM_009388204.2	Nucleus (0.6779)	Cytoplasm (0.650)	Nucleus (0.60)	Other (0.9998)	Cytoplasm/nucleus	Nucleus
MusaBAG5	XM_009412890.2	Cytoplasm (0.4668)	Mitochondrial matrix space (0.666)	Chloroplast (0.13)	Other (0.9921)	Chloroplast	Nucleus
MusaBAG6	XM_009422955.2	Nucleus (0.5599)	Nucleus (0.700)	Nucleus (0.20)	Other (0.9773)	Nucleus	Nucleus
MusaBAG7	XM_009387694.2	Nucleus (0.7599)	Nucleus (0.700)	Cytoplasm (0.18)	Other (0.9997)	Cytoplasm	Chloroplast
MusaBAG7	XM_009390167.2	Nucleus (0.824)	Microbody (0.328)	Nucleus (0.09)	Other (0.9999)	Cytoplasm	Chloroplast
MusaBAG8	XM_009400601.2	Nucleus (0.6567)	Microbody/nucleus (0.300)	Nucleus (0.07)	Other (0.9995)	Nucleus	Nucleus
MusaBAG8	XM_009399476.2	Plastid (0.5135)	Nucleus (0.300)	Nucleus (0.70)	Other (0.9996)	Nucleus	Chloroplast and Nucleus
MusaBAG8	XM_018825107.1	Nucleus (0.4769)	Microbody (0.657)	Nucleus (0.13)	Other (0.9962)	Nucleus	Nucleus

### Putative cis-element in promoter sequences

The conserved *cis*-regulatory element present in the promoter regions are known to be the binding sites for several transcription factors, that in turn regulate the spatial and temporal transcriptional activity. A large number of such conserved *cis*-elements were identified in the putative promoter sequences of *MusaBAG* genes when queried in the PlantCARE and PlantPAN databases. Multiple stress-related, light responsive and hormonal regulated *cis*-elements were identified in the promoter regions of *MusaBAG* genes (Table 3). Elements related to defense responses (STRE, WRKY and TC-rich repeats), drought (DRE, MBS, LIM), low temperature (LTR), wounding (W-box, WUN-motif and WRE3) and anaerobiosis (AREs) were observed. Many light-responsive elements such as LAMP, GT1, GATA motif, ATCT, Sp1, G Box, AE-box, Box 4, Box II Chs-CMA1a, ACE and I Box were present suggesting role of MusaBAG proteins in regulation of photoperiodic control and development. Moreover, binding sites for transcription factor involved in stress responses, growth and development were also identified in the promoter region that includes Myb, bZIP, HSF, WRKY, AP2, C2H2, C3H, AT-Hook, bHLH, SBP, Dof, NAC, WOX, CPP, Storekeeper, nuclear factor-Y (NF-Y), GRAS, LFY, VOZ, YABBY, MADS, TCP, ARID, TALE and Homeobox (HD-ZIP). Furthermore, hormone specific *cis*-regulatory elements like ABRE (abscisic acid), P-box (gibberellin), ERE (ethylene), AuxRR-core (auxin), GARE-motif (gibberellin), TGA-element (auxin, salicyclic acid), ARF (auxin), MYC (auxin), EIN3 (ethylene), ERF (ethylene), BES1 (brassinosteroids) and CGTCA (methyl jasmonate) were present. AC-I and AC-II are involved in xylem specific expression whereas O2-site has a role in circadian control. Few sugar responsive elements such as SRS and α-amylase box were also identified in the promoter sequences of *MusaBAG* genes. Predominance of a variety of binding sites for transcription factors involved in stress and plant hormonal responses in the *MusaBAG* genes promoter region clearly indicate its role in essential biological processes such as stress tolerance and plant growth and development.

**Table 3:** *Cis*-regulatory elements present in the putative promoter sequences of the *MusaBAG* genes.

Name	Accession	PlantPAN 3.0	PlantCARE
BAG family molecular chaperone regulator 1-like	XM_009400456.2	AT-Hook, NAC;NAM, MYB; ARR-B, AP2;ERF, bZIP, Dof, B2;ARF’ bHLH, CSD, CG-1, EIN3, GATA;tify, HD-ZIP, WOX, MADF, MYB;SANT, SBP, SOX;YABBY, TCP, Alpha-amylase, WRKY, MADS Box, LFY, zF-HD, NF-YA, NF-YB, MF-YC, LEA_5, ARID, BES1, C2H2, C3H, FAR1, VOZ	ABRE, AE-box, AuxRR-core, Box 4, Box II, DRE core, G-Box, HD-Zip 1, LTR, MYB, MYB recognition site, MYC, Sp1, TC-rich repeats, TCA-element, TGA-box, W box, WRE3, as-1
BAG family molecular chaperone regulator 1-like	XM_009409679.2	AT-Hook, NAC;NAM, MYB;ARR-B, AP2;ERF, C2H2, bZIP, AP2;ERF, B3;ARF, GATA;tify, MYB;G2like, LOB;LBD, trp, Dof, bHLH, bZIP, C2H2, CG-1;CAMTA, CSD, TCR;CPP, E2F/DP;E2F, EIN3;EIL, GRAS, HD-ZIP, MADF, Myb/SANT, Myb/SANT;G2-like, Myb/SANT;ARR-B; ARR-B, SBP, Sox;YABBY, Storekeeper;GeBP, TBP, TCP, WRKY, MADS box, B3, C2H2, LEA type 1, HB-PHD, VOZ, ZF-HD, SBP, TALE;TALE, NF-YB;NF-YA;NF-YC, Dehydrin, Bet_v_1, ERF, WOX, Alpha-amylase, B3, ARID;Sox, BBR-BPC, C3H, mTERF	A-box, ABRE, ARE, Box 4, G-box, GATA-motif, I-box, LTR, MBS, MYB recognition site, MYC, Myb, Sp1, W box, WRE3, as-1
BAG family molecular chaperone regulator 1	XM_009393675.2	AT-Hook, NAC;NAM, MYB;ARR-B, AP2;ERF, C2H2, bZIP, Dof, B3;ARF, GATA;tify, AP2;RAV, AP2;ERF;ERF, LIM, HD-ZIP, AP2;B3;RAV, bHLH, CG-1;CAMTA, CSD, TCR;CPP, E2F/DP;E2F, EIN3;EIL;EIL, MADS box;MIKC, GRAS, TALE;TALE, ZF-HD;ZF-HD, MADF, Myb/SANT;G2-like, Myb/SANT;MYB;ARR-B, SBP, Storekeeper;GeBP, TCP, WRKY, BES1, FAR1, MADS box, LEA type 1, Alpha-amylase, VOZ, ZF-HD, NF-YB;NF-YA;NF-YC, B3, Ribosomal protein L21P, SRS, Aux/IAA, E2F/DP, ARID, BBR-BPC, C2C2COlike, C2H2, C3H Zinc finger, TCR;CPP, EIN3;EIL, WOX, Sox, LOB; LBD, mTERF,	A-box, ABRE, ATC-motif, DRE1, ERE, G-box, GT1-motif MYB recognition site, MYC, Myb, O2 site, OCT, Sp1, W box, WUN-motif, as-1
BAG family molecular chaperone regulator 1-like	XM_009394078.2	AT-Hook, NAC;NAM, MYB;ARR-B, Myb/SANT;MYB;ARR-B, AP2;ERF, C2H2, bZIP, AP2, B3;ARF, GATA;tify, MYB;G2-like, LOB;LBD, AP2;RAV, AP2;ERF;ERF, trp, Dof, bHLH, AP2;B3, E2F/DP;E2F, EIN3;EIL;EIL, TALE;TALE, HD-ZIP, MADF, Storekeeper;GeBP, TBP, TCP, MADS box;MIKC, LFY, LIM, MADS box, HSF, Alpha-amylase, HB-PHD, SBP, ZF-HD, NF-YB;NF-YA;NF-YC, Dehydrin, WRKY, SRS, LEA_5, B3, ARID;Sox, BBR-BPC, BES1, YABBY, E2F/DP, EIN3;EIL,	ABRE, ABRE2, ABRE3a, ABRE4, Box 4, Box II, AC-I, AC-II, ACE, AE-Box, DRE core, G-box, GARE-motif, LTR, MBS, MYB recognition site, MYC, Myb, NON Box, O2 site, Sp1, WRE3, as-1
BAG family molecular chaperone regulator 2-like	XM_018825077.1	AT-Hook, MYB;ARR-B, AP2;ERF, C2H2, bZIP, HD-ZIP, Dof, B3-RF, MYB;G2-like, AP2;RAV, bHLH, AP2, TCR;CPP, EIN3;EIL, GATA;tify, GATA;Dof, GRAS, TALE;TALE, HD-PHD, ZF-HD, WOX, MADF, MYB-SANT, SBP, Sox;YABBY, Storekeeper, TBP, TCP, WRKY, MADS Box, BES1, YABBY, alpha-amylase, LEA-type I, HSF, PsaH, HB-PHD, NF-YB;NF-YA;NF-YC, Dehydrin, Ribosomal protein L21P, Aux/IAA, ARID;Sox, C3H zinc finger, CAMTA, WRC;GRF, LOB;LBD, MADS box;MIKC	ABRE, ABRE2, AE-Box, ARE, ATC-motif, ATCT-motif, AuxRR-core, Box 4, Box II, DRE core, G-box, GARE-motif, MBS, MYB recognition site, Myb, LAMP element, GT1 motif, O2 site, STRE, W Box, Box S, as-1
BAG family molecular chaperone regulator 4	XM_009388204.2	AT-Hook, AP2;ERF, C2H2, bZIP, HD-ZIP, Dof, B3-ARF, MYB;G2-like, trp, AP2;B3;RAV, bHLH, bZIP, CSD, MYB/SANT;MYB-related, AP2, TCR;CPP, EIN3;EIL, GATA;tify, GATA;Dof, Storekeeper;GeBP, GRAS, TALE;TALE, HD-PHD, ZF-HD, WOX, MADF, SBP, Sox;YABBY, TBP, TCP, WRKY, MADS Box, BES1, Sox;YABBY, alpha-amylase, LEA-type I, Bet_v_1, LEA_5, E2F/DP, HSF, PsaH, HB-PHD, NF-YB;NF-YA;NF-YC, Dehydrin, Ribosomal protein L21P, Aux/IAA, ARID;Sox, C2C2COlike, C3H zinc finger, VOZ, WRC;GRF, LOB;LBD, MADS box;MIKC	ABRE, AC-I, AC-II, ARE, AT1-motif, AuxRR-core, Box 4, ERE, G-box, GARE-motif, GC-motif, GT1-motif, I-box, MBS, MYB, MYB recognition site, MYB-like sequence, MYC, Myb-binding site, P-box, STRE, TCA, TCT-motif, TGA-box, TGACG-motif, Wbox, WRE3, as-1, dOCT
BAG family molecular chaperone regulator 5	XM_009412890.2	AT-Hook, NAC; NAM, AP2; ERF, bZIP, MYB-related, Dof, B3; ARF, GATA; tify, MYB; G2-like, AP2; RAV, HD-ZIP, C2H2, CSD, TCR; CPP, EIN3; EIL, TALE, Myb/SANT; ARR-B, Sox;YABBY, Storekeeper, TBP, TCP, WRKY, MADS box; MIKC, HB-PHD, VOZ, ZF-HD, SBP, NF-YB;NF-YA;NF-YC, Dehydrin, bHLH, WOX, ARID; Sox, BES1, YABBY, C3H Zinc finger, TCR; CPP, GeBP, HSF, WRC; GRF, LOB; LBD, SRS,	A-box, ABRE, ABRE3a, ABRE4, AT ~ TATA-box, Box 4, CGTCA-motif, ERE, G-box, GATA-motif, GCN4_motif, LTR, MBS, MYB, MYB-like sequence, MYC, Myb, Myb-binding site, O2-site, STRE, Sp1, TCA, TCT-motif, W box, WRE3, as-1, chs-CMA1a
BAG family molecular chaperone regulator 6-like	XM_009422955.2	AT-Hook, NAC;NAM, MYB;ARR-B, bZIP;HD-ZIP, MYB-related, B3;ARF, GATA;tify, MYB;G2-like, trp, AP2;B3;RAV, Dof, bHLH, C2H2, TCR;CPP, EIN3;EIL;EIL, MADS box;MIKC, GRAS, TALE;TALE, HB-PHD, WOX;WOX, LOB;LBD;LBD, MADF;Trihelix, SBP, Storekeeper;GeBP, TBP, TCP, WRKY, MADS box;MIKC; M-type, SRS, HSF, Dehydrin, Alpha-amylase, NF-YB;NF-YA;NF-YC, CG-1;CAMTA, BES1, Bet_v_1, WOX, LEA_5, ARID;Sox, BBR-BPC,	AAGAA-motif, ACE, ARE, AuxRR-core, G-box, GATA-motif, GT1-motif, Gap-box, LAMP-element, MYB, MYB recognition site, MYB-like sequence, MYC, Myb-binding site, Myc, P-box, STRE, TCA-element, TCT-motif, TGA-element, W box, WRE3
BAG family molecular chaperone regulator 7-like	XM_009387694.2	AT-Hook, NAC;NAM, C2H2, bZIP, MYB;G2-like, AP2;ERF;ERF, trp, Dof, LIM, AP2;B3; RAV, bHLH, CG-1;CAMTA;CAMTA, CSD, TCR;CPP, GATA;tify, GRAS, WOX;WOX, MADF;Trihelix, Myb/SANT;MYB-related, SBP, Sox;YABBY, TBP, TCP, WRC;GRF, WRKY, BES1, MADS box;MIKC, LIM, SRS, HSF, HB-PHD, VOZ, ZF-HD, NF-YB;NF-YA;NF-YC, Dehydrin, Alpha-amylase, Bet_v_1, Ribosomal protein L21P, ARID;Sox, E2F	3-AF1 binding site, A-box, AAGAA-motif, ABRE, ABRE3a, ABRE4, ARE, Box 4, Box III, CCGTCC motif, CCGTCC-box, CGTCA-motif, G-Box, GARE-motif, GATA-motif, GT1-motif, LAMP-element, MBS, MYB, MYB-like sequence, MYC, Myb-binding site, O2-site, STRE, TCA-element, TGA-element, TGACG-motif, W box, as-1, box S
BAG family molecular chaperone regulator 7-like	XM_009390167.2	AT-Hook, NAC; NAM, MYB;ARR-B, Myb/SANT, C2H2, bZIP, MYB-related, GATA;tify, MYB;G2-like, AP2;RAV, AP2;ERF, bHLH, B3;ARF, CG-1;CAMTA, CSD, TCR;CPP, Dof, EIN3;EIL, HD-ZIP, HB-PHD, WOX, LOB;LBD, MADF;Trihelix, Sox;YABBY, TBP, TCP, WRC;GRF, WRKY, BES1, FAR1, bHLH, SBP, B3, SRS, YABBY, HSF, VOZ, NF-YB;NF-YA;NF-YC, Dehydrin, ARID;Sox, C3H Zinc finger, CG-1;CAMTA, E2F/DP, EIN3;EIL, LOB;LBD, ZF-HD, GeBP, TALE	ABRE, ABRE3a, ABRE4, ARE, AT-rich sequence, AuxRE, Box 4, CGTCA-motif, DRE core, G-Box, G-box, GATA-motif, GT1-motif, GTGGC-motif, MYB recognition site, MYC, Myc, O2-site, P-box, STRE, TC-rich repeats, TCA, TCA-element, TGA-element, TGACG-motif, W box, WUN-motif, as-1, chs-Unit 1 m1
Uncharacterized LOC103983382, (BAG8)	XM_009400601.2	AT-Hook, MYB;ARR-B, Myb/SANT, AP2;ERF, C2H2, bZIP, B3;ARF, MYB;G2-like, LOB;LBD, AP2;RAV, bHLH, AP2;B3, bZIP, TCR;CPP, CG-1;CAMTA, Dof, EIN3;EIL, GATA;tify, HD-ZIP, MADF, NAC;NAM, Sox;YABBY, Storekeeper, TCP, WRKY, BES1, MADS box;MIKC, SBP, HB-PHD, NF-YB;NF-YA;NF-YC, Dehydrin, Trihelix, ARID;Sox, GeBP, GRF, WOX, HSF, LOB;LBD,	A-box, ABRE, AE-box, Box 4, CCGTCC motif, CGTCA-motif, DRE core, F-box, G-box, GARE-motif, GATA-motif, GC-motif, GCN4_motif, LAMP-element, LTR, MBS, MRE, MYB, MYB recognition site, MYB-like sequence, MYC, Myb, Myb-binding site, O2-site, P-box, STRE, Sp1, TCCC-motif, TGACG-motif, WRE3, WUN-motif, as-1, box S
BAG family molecular chaperone regulator 8, chloroplastic	XM_009399476.2	bZIP, LOB;LBD, AP2;ERF;ERF, trp, bHLH, bZIP, C2H2, TALE;TALE, EIN3;EIL;EIL, MADF;Trihelix, MADF, Myb/SANT, NAC;NAM, SBP, TCP, WRKY, BES1, HB-PHD, Alpha-amylase, VOZ, GATA;tify, Dof, ZF-HD, B3, NF-YB;NF-YA;NF-YC, Dehydrin, Trihelix, Ribosomal protein L21P, Alpha-amylase, C3H Zinc finger, C3H, GRF	A-box, ABRE, CCGTCC motif, CCGTCC-box, CGTCA-motif, DRE core, G-box, MBS, MYB, MYB-like sequence, MYC, Myb, O2-site, STRE, Sp1, TGACG-motif, WRE3, as-1

### Digital expression patterns and ESTs

**Figure 6:**
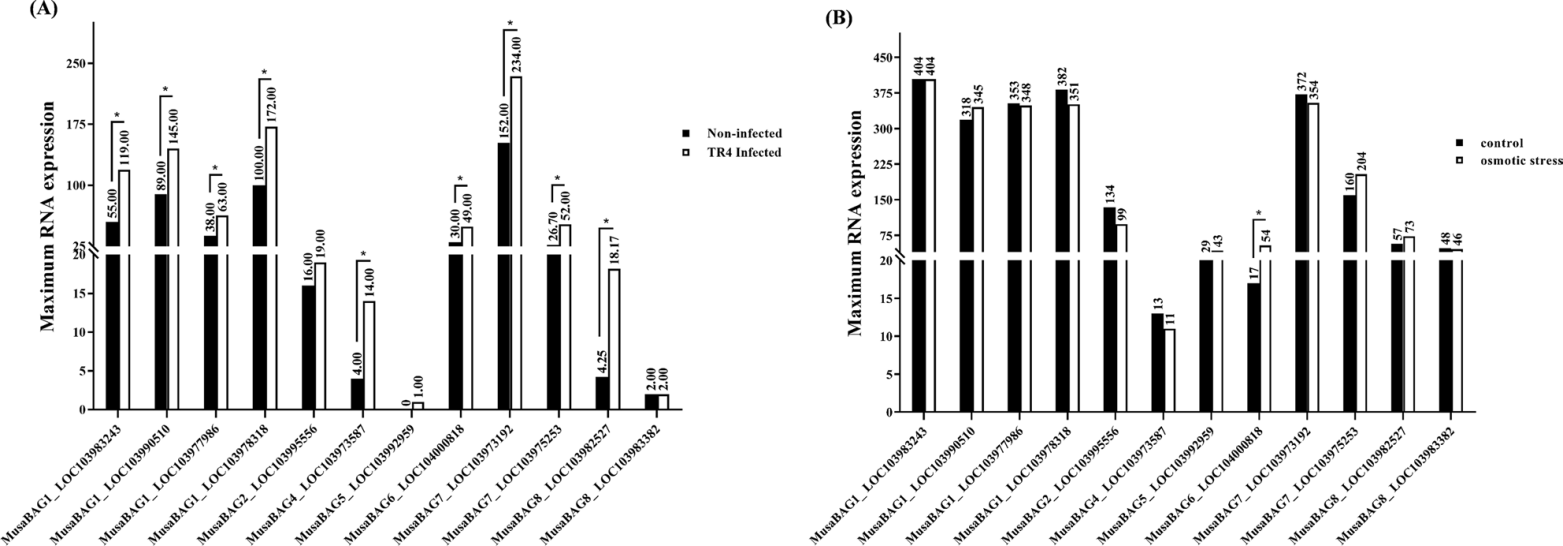
Relative expression profile of *MusaBAG* genes in biotic and abiotic stresses using the RNA-seq data of *Musa acuminata* DH-Pahang (AA) available in the banana genome hub. (**A**) Expression levels of *MusaBAG* genes inoculated with *Fusarium oxysporum* f. sp. *cubense* tropical race 4 (Foc TR4, now *Fusarium odoratissimum*) depicted in white bars with respect to the uninfected control (depicted as black bars) showed significant upregulation of *MusaBAG1, 4, 6, 7* and *8* at P < 0.05 (**B**) The expression levels of *MusaBAG* genes under osmotic stress condition (depicted as white bars) showed no significant difference except *MusaBAG6* at P < 0.05, as compared to the unstressed control plants (depicted as black bars).

The maximum expression of mRNA for different *MusaBAG*s were analyzed from the RNAseq data available in banana genome hub for *Musa acuminata* DH Pahang variety (under control or non-infected condition and Foc TR4 infected condition; Fig. 6A) and Cavendish Grand Naine variety under control and osmotic stress condition in the roots (Fig. 6B). There was a significant difference observed in the expression of *MusaBAG* genes (P < 0.05, two-tailed non parametric t-test), under Foc TR4 infection whereas no significant difference was observed under osmotic stress, as compared to the respective control conditions. Amongst all, *MusaBAG2, MusaBAG5* and the truncated variant of *MusaBAG8* (LOC103983382) did not show a significant change in the expression levels under Foc TR4 infected and non-infected conditions. Two out of the four *MusaBAG1* genes (LOC103983243, LOC103978318) and one of the *MusaBAG7* (LOC103975253) showed onefold overexpression under Foc TR4 infected condition as compared to the non-infected control. The remaining two *MusaBAG1* (LOC103990510, LOC103977986), *MusaBAG6* and *MusaBAG7* (LOC103973192) showed 1.5 times expression under Foc TR4 infected condition. *MusaBAG4*, and *8* displayed around twofold expression under infected condition, as compared to the non-infected condition (Fig. 6A). In contrast, no such difference in the expression of *MusaBAG* genes was observed under osmotic stress condition, when compared with respective root osmotic control. However, only *MusaBAG6* mRNA expression showed 1.5 fold overexpression, as compared to the control (Fig. 6B). Based on the Sidak’s multiple comparison test of hypothesis (P < 0.05), significant difference was observed in *MusaBAG1, 4, 6, 7 and 8* genes under biotic stress conditions and *MusaBAG6* gene under abiotic stress condition, with respect to their controls.

### Expression analysis of MusaBAG genes

**Figure 7:**
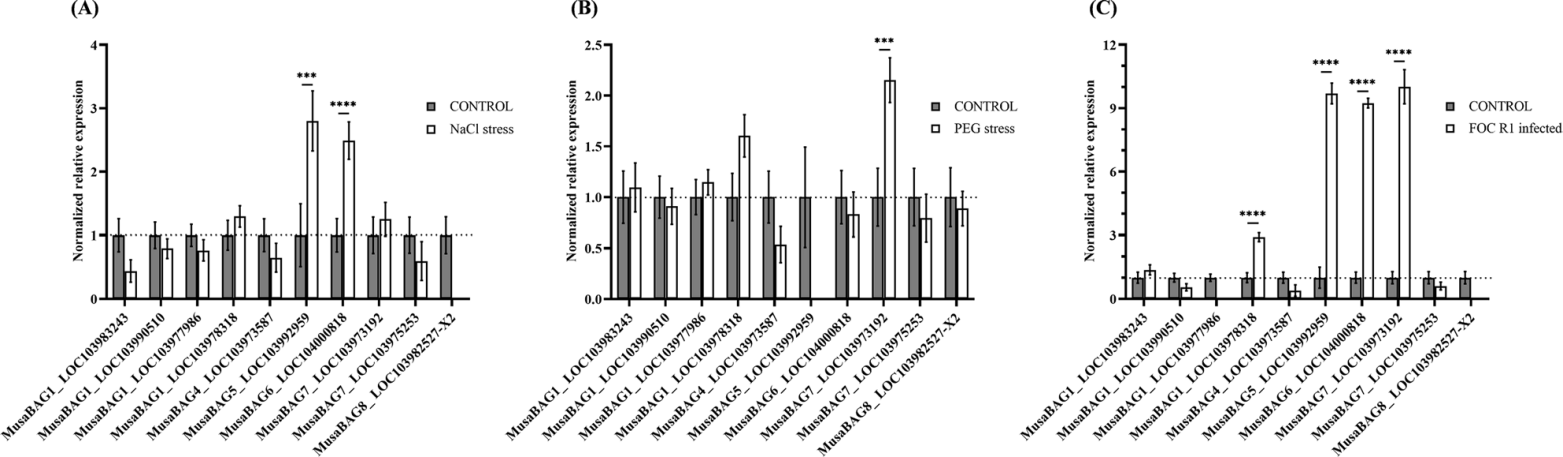
Relative expression profile of *MusaBAG* genes in biotic and abiotic stress condition using quantitative RT-PCR analysis. (**A**) The expression levels of *MusaBAG* genes under sodium chloride stress condition (depicted as white bars) showed significant difference for *MusaBAG5* as well as *MusaBAG6* at P < 0.001 and P < 0.0001 respectively, as compared to the unstressed control plants (depicted as grey bars). (**B**) The expression levels of *MusaBAG* genes under PEG stress condition (depicted as white bars) showed significant difference only for *MusaBAG7*_LOC103973192 at P < 0.001, as compared to the unstressed control plants (depicted as grey bars). (**C**) Expression levels of *MusaBAG* genes inoculated with *Fusarium oxysporum* f. sp. *cubense* race 1 (Foc R1) depicted in white bars with respect to the uninfected control (depicted as grey bars) showed significant upregulation of *MusaBAG1*_LOC103978318, *MusaBAG5*, *MusaBAG6* and *MusaBAG7*_LOC103973192 at P < 0.0001.

Given the fact that plants initiate a protective response during stress conditions so as to maintain cellular homeostasis, genes like *MusaBAG* seem to play an important role. To ascertain the importance of *MusaBAG* genes we studied their differential regulation in banana plants exposed to various abiotic and biotic stress conditions. Overall there was a significant difference in the expression pattern of *MusaBAG1*_LOC103978318, *MusaBAG5*_LOC103992959, *MusaBAG6*_LOC104000818 and *MusaBAG7*_LOC103973192 under abiotic and biotic stress conditions as compared to the unstressed control (Fig. 7). In case of sodium chloride stress, *MusaBAG5*_LOC103992959 and *MusaBAG6*_LOC104000818 were upregulated 3 and 2.5 times, respectively; whereas *MusaBAG8*_LOC103982527-X2 remains undetected in the treated samples (Fig. 7A). However, only *MusaBAG7*_ LOC103973192 was found to be upregulated 2 times in PEG induced stress and *MusaBAG5*_LOC103992959 remains undetected (Fig. 7B). In biotic stress, the *Fusarium oxysporum* f. sp. *cubense* race 1 (Foc R1) infected plants showed 3 times upregulation of *MusaBAG1*_LOC103978318 whereas, *MusaBAG5*_LOC103992959, *MusaBAG6*_LOC104000818 and *MusaBAG7*_LOC103973192 were upregulated around 10 times (Fig. 7C). Further, no amplification was observed for *MusaBAG1*_LOC103977986 and *MusaBAG8*_LOC103982527-X2 under this condition. Furthermore, under this experimental setup no detectable expression was observed for *MusaBAG2*, *MusaBAG8*_LOC103983382, *MusaBAG8*_LOC103982527-X1 in any of the treated or control plants.

### Protein–protein interactions

**Figure 8:**
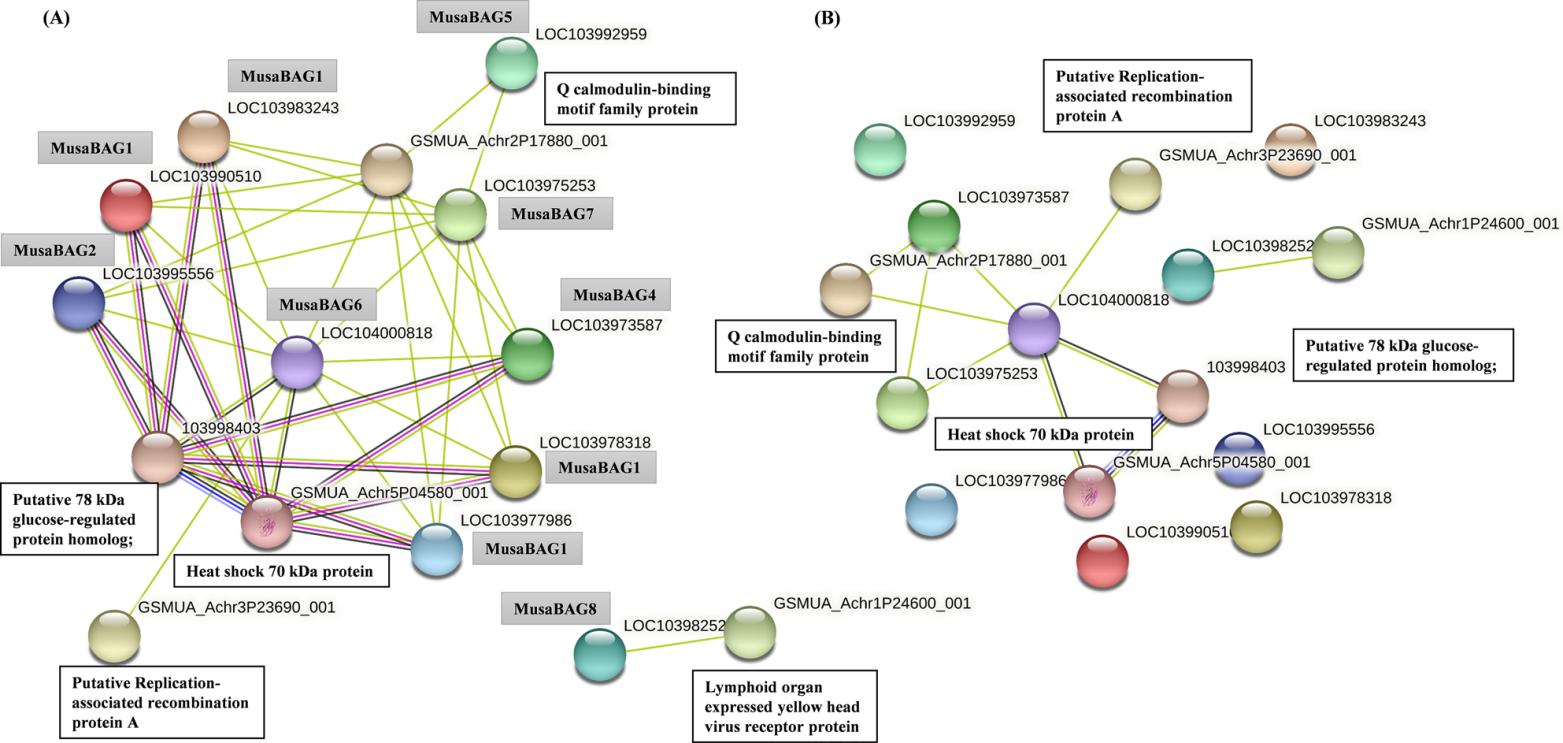
Protein–protein interaction network of MusaBAG proteins. The network was generated using STRING version 11.0, where each node represents a protein and each connecting line represents an interaction. The green line indicates interaction established through textmining, blue indicates co-occurrence of the proteins whereas pink link denotes interaction established through experimental evidence with moderate (**A**, 0.400) and high (**B**, 0.700) confidence levels.

BAG proteins are known to carry out their function by interacting with other proteins. So to identify the potential interactors of MusaBAG proteins the interacting partners for each MusaBAG protein was searched using the STRING database version 11 with medium confidence of 0.400 (Supplementary Fig. S2). The predicted interactome was mainly based on textmining, coexpression and experimental data. All the MusaBAG1 paralogs and MusaBAG2 had common interacting proteins (Supplementary Fig. S2A). The potential interactors include uncharacterized GPI-anchored protein, putative lipoxygenase homology domain containing protein 1, Tra family proteins and IQ-calmodulin-binding motif family protein. Moreover, MusaBAG1 and MusaBAG2 were shown to be interacting with the autophagy related proteins through the ubiquitin-associated protein. MusaBAG4 showed a more complex network of interactome (Supplementary Fig. S2B). The direct interactors of MusaBAG4 were vacuolar protein sorting-associated protein 32 homolog 1, IQ-calmodulin-binding motif family protein and putative ubiquitin associated protein and indirectly interacted with putative ALG-2 interacting protein X. Similar to MusaBAG1 and MusaBAG2, MusaBAG4 also showed interaction with autophagy related proteins through the ubiquitin associated protein. Through textmining, MusaBAG5 was predicted to be interacting with IQ-calmodulin-binding motif family protein and an uncharacterized protein which in turn were predicted to interact with several other proteins such as luminal binding proteins, BAG domain proteins and other uncharacterized proteins (Supplementary Fig. S2C). MusaBAG6 showed a complex network of proteins forming distinct clusters involved in DNA processing, heat shock proteins and proteinases (Supplementary Fig. S2D). MusaBAG6 was predicted to be interacting with another BAG domain containing proteins and several heat shock proteins. It was predicted to be interacting with putative replication associated recombination protein A which further interacted with three other protein domains namely, DNA polymerase delta catalytic subunit, DNA helicase and putative protein BREAST CANCER SUSCEPTIBILITY 1 homolog. MusaBAG7 paralogs were predicted to be interacting with luminal binding proteins (sensors of endoplasmic stress), heat shock protein binding proteins and calnexin homolog 1 (Supplementary Fig. S2E). Moreover, an indirect interaction with other BAG domain proteins was also predicted for MusaBAG7. The interactors for MusaBAG8 were very different from the other MusaBAG proteins. MusaBAG8 interactors were predicted based on textmining and showed lymphoid organ expressed yellow head virus receptor protein, growth regulator related protein, HEAT repeat family proteins, EPS4 proteins and STE_PAK_STE20_MST__like_1-STE kinase homologs to sterile 7 as potential interacting proteins (Supplementary Fig. S2F). Through this network, MusaBAG8 was also predicted to be interacting with other proteins indirectly that included, heat shock proteins, putative calcium binding protein 32 like, zinc finger CCCH domain containing protein 45 and cleavage and polyadenylation specificity factors. Among the MusaBAG proteins, all of them were predicted to be interacting with each other except for MusaBAG8 (Fig. 8). At high confidence (0.700) in this interactome strong interaction was predicted between MusaBAG6, Heat shock 70 kDa protein, putative replication associated recombination protein A and putative 78 kDa glucose-regulated protein homolog (Fig. 8). Through textmining at a high confidence level an interaction between MusaBAG4, MusaBAG7, and MusaBAG6 was predicted.

## Discussion

The *BAG* gene family encodes for a multifunctional group of proteins that regulate diverse pathways such as cell signaling, stress responses (both abiotic and biotic), growth and development^1^. BAG proteins were acknowledged to be the critical regulators of PCD in plants and their importance in maintaining homeostasis was established. Inadequacy in the knowledge of plant PCD as compared to that in animals have always been a limitation in engineering improved traits in crop plants. Since BAG proteins seem to be the core regulators, understanding this network and pathway will aid in modulating plant PCD towards environmental stimuli (both abiotic and biotic). In this study we carried out a genome wide search to identify *BAG* genes in an important fruit crop, banana using *Arabidopsis BAG* genes as query sequences. Banana is sensitive to extremes of environmental conditions and cannot sustain under drought, high salinity and low temperature^16,17^. Banana plants are also susceptible to many pests and diseases that cause severe yield losses^18^. In all these events PCD seems to play a crucial role, and thus studying *BAG* genes in banana plants is warranted. A total of 13 *MusaBAG* genes were identified in the whole genome of banana (*Musa acuminata*) dispersed on seven out of the eleven chromosomes; namely *MusaBAG1* (4 paralogs), *MusaBAG2*, *MusaBAG4*, *MusaBAG5*, *MusaBAG6*, *MusaBAG7* (2 paralogs) and *MusaBAG8* (1 paralog and 2 transcript variants). Multiple members of *MusaBAG* genes in the banana genome thus reveal the importance and diverse role of these genes in various cellular functions.

All plant BAG proteins contain the typical BAG domain located towards the C terminus that interacts with the ATPase domain of the HSP70 protein required for its co-chaperone activity^7^. All the MusaBAG proteins showed the presence of this characteristic BAG domain except MusaBAG8 (LOC103983382). In addition, MusaBAG1, MusaBAG2 and MusaBAG4 contain ubiquitin-like domain for recruitment of proteasome degradation machinery for the degradation of misfolded and unimported chloroplast proteins in the cytosol^11,19^. The ubiquitin-like domain present in MusaBAG1, 2 and 4 is the site of ubiquitination by E1, E2 and E3 ligases that eventually results in degradation either through proteasomal degradation or autophagy^20^. An E3 ubiquitin ligase, enhanced blight and blast resistance 1 (EBR1), interacts with rice OsBAG4 protein having ubiquitin-like domain targeting it for ubiquitination-mediated degradation resulting in susceptibility to *Xanthomonas oryzae* pv. *oryzae* and *Magnaporthe oryzae*^19^. MusaBAG5, MusaBAG6, MusaBAG7 and MusaBAG8 contain CaM-binding motif, named as IQ motif suggesting its role in the calcium-calmodulin signaling pathway. This IQ motif present in the BAG proteins preferentially binds the calcium-free state of CaM. In the presence of calcium, there is release of Hsc70 due to competitive binding between CaM and Hsc70 to the AtBAG5 eventually preventing PCD in *Arabidopsis* plants^10^. Presence of the KED sequence motif in MusaBAG7 indicates its importance in wound healing and maintaining homeostasis during cytosolic acidification created through MAP kinase and defense gene activation^21^. The electron density map of this motif shows a disordered hand domain-like structure having interaction pockets similar to that of the EF-hand proteins, suggesting possible roles in metal (Ca^2+^ and Mg^2+^) binding^22^. The predicted structures of MusaBAG proteins displayed a typical BAG domain consisting of a three-helix bundle. Helices 2 and 3 contact subdomains IB and IIB of the ATPase mainly utilizing residues Glu212, Asp222, Arg237, and Gln245 of mammalian BAG1 protein^15^. Arg237 is highly conserved in all known BAG proteins, and their individual replacement with alanine resulted in BAG variants with substantially decreased ATPase activity and reduced affinities for the Hsc70 ATPase domain^15,23^. The relatedness among the MusaBAGs and homologs from other species was depicted using a phylogenetic tree and showed similarity between these protein sequences arranged together in different clades. The presence of paralogs on the same chromosome indicates the possibility of a tandem duplication event whereas those on different chromosomes are possibly due to segmental duplication events. However, multiple copies and duplication of genes with conserved or additional domains increases the functional divergence of these proteins and establishes its role in the important function of adaptability under changing environmental conditions.

Putative *cis*-acting elements related to light, stress, hormone and development were identified in the promoter regions of *MusaBAG* genes. The presence of these elements also indicate that they could be regulated by abiotic and biotic stress and different hormones. The *MusaBAG* genes could be regulated under stress conditions due to the presence of conserved stress-responsive *cis*-elements, such as DRE, ABRE, MYC, HSE, and MYB-binding sites. These genes also seem to be regulated by several transcription factors such as bZIP, WRKY, NAC and bHLH that are known to be triggered during abiotic stress and attack by pests or pathogens^24–27^. These results suggest that *MusaBAG* genes are regulated by multiple mechanisms and have functional divergence. Li et al.^8^ showed that AtBAG6 is cleaved by caspase-like proteases and triggers autophagy in the host preventing *Botrytis cinerea* infection and confers basal resistance. Expression of *BAG1* (BAG family molecular chaperone regulator 1-like) was also differentially regulated in banana cells upon *Fusarium* inoculation and toxins under in vitro conditions and further overexpression of this *BAG1* in banana plants imparted resistance to Fusarium wilt disease^13^. *BAG* genes are also differentially regulated under abiotic stress conditions and provide protection against extremes of conditions. Overexpression of *AtBAG4* in tobacco plants demonstrated tolerance to abiotic stresses such as UV light, cold, oxidants, and salt treatments^3^. In our study, we observed differential regulation of some of the *MusaBAG* genes under abiotic and biotic stress conditions. *MusaBAG5*_LOC103992959 and *MusaBAG6*_LOC104000818 were upregulated in sodium chloride induced stress; whereas *MusaBAG7*_LOC103973192 was found to be upregulated in PEG induced stress. Significant upregulation of *MusaBAG1*_LOC103978318 *MusaBAG5*_LOC103992959, *MusaBAG6*_LOC104000818 and *MusaBAG7*_LOC103973192 was observed in banana plants infected with Foc R1. The total number of *MusaBAG* genes upregulated in the Foc R1 resistant cultivar Grand Naine as per the RNAseq data was higher in comparison to the gene expression profile obtained in the susceptible cultivar (Rasthali) indicating its role in resistance response. Noteworthy upregulation seen in the MusaBAG genes in Foc R1 treated Rasthali plants indicate that they are undergoing immense stress and have induced protective mechanisms involving these genes. Under abiotic stress, *MusaBAG*5, *MusaBAG6* and *MusaBAG7* genes seems to play a critical role. However, further investigations are required to better understand the role of these *MusaBAG* genes in early or late and susceptible or resistant responses in banana plants.

Each of these MusaBAG proteins are also compartmentalized in the cell for their specialized function. Most of the MusaBAG proteins were predicted to be located in the nucleus, mitochondria and chloroplast, MusaBAG6 was predicted to be localized in the nucleus. At their respective destinations MusaBAG proteins interact with their partners to carry out their specific functions. MusaBAG1, MusaBAG2 and MusaBAG4 interact with the autophagy-related proteins through the UBQ domain for protein degradation. MusaBAG4 also interacts with the vacuolar protein sorting associated proteins hinting at its role in endosomal sorting complexes required for transport of proteins to the vacuoles^28,29^. MusaBAG5 and MusaBAG7 localized in the mitochondria, chloroplast or microbody were shown to interact with the luminal binding proteins (BiP). Plant BiP possesses molecular chaperone activity targeting protein folding and maturation^30^. Overexpression of BiP in transgenic plants resulted in abiotic stress tolerance phenotype^31^. In previous studies, the *Arabidopsis* homolog of BAG7, has been shown to have interaction with several ER-stress related transcription factors, such as bZIP and WRKY29, and later translocated to the nucleus^25^. In our study, MusaBAG6 was shown to interact with various DNA binding proteins and previous studies demonstrated its role in cell death pathways associated with abiotic stress conditions^3,7,32^. MusaBAG8 was predicted to be interacting with a unique set of proteins which were uncommon among the other MusaBAG proteins. Thus MusaBAG8 probably has diversified a lot and now is involved in other crucial unknown functions. Extensive functional studies are needed to ascertain the role of each of these *BAG* genes. It will be of considerable interest to experimentally validate the protein interactors of MusaBAG and to characterize the pathways involved in stress tolerance.

In conclusion, BAG proteins are an important link between molecular chaperones and protein degradation pathways (proteasome or autophagy) that maintain cellular homeostasis. The banana *BAG* gene family can be explored to understand their role in growth, development, fruit ripening, and fundamental responses to abiotic and biotic stresses. This knowledge can then be extended to other horticulture plants to understand the regulation of BAG proteins and exploit them in crop improvement programs.

## Methods

### Musa database search for BAG domain proteins

The full-length coding sequences of *AtBAG*s^6^ were retrieved from NCBI database (https://blast.ncbi.nlm.nih.gov/Blast.cgi)^33^ and were used to identify their respective homologs in the banana genome (*Musa acuminata, *https://banana-genome-hub.southgreen.fr/)^34^ denoted as *MusaBAG*s. The copy number of each of the *MusaBAG*s was determined by performing a nucleotide BLAST in the banana genome hub (https://banana-genome-hub.southgreen.fr/)^34^. Each of the *MusaBAG* genes were mapped on the 11 chromosomes (A genome) of *Musa acuminata* DH-Pahang (AA) usingMap2GeneChrom v2.1 tool (http://mg2c.iask.in)^35^. The full-length coding sequence and protein sequence of *MusaBAG* genes were also retrieved from the NCBI database (https://blast.ncbi.nlm.nih.gov/Blast.cgi) for further analysis^33^. The molecular weight and the isoelectric point of these proteins were analyzed using the ExPASy online tool (https://web.expasy.org/compute_pi/)^36^. The *BAG* genes of rice denoted as the *OsBAG*s were also retrieved from the NCBI database (https://blast.ncbi.nlm.nih.gov/Blast.cgi)^33^ using the accession number mentioned in previous studies^37^.

### Conserved domain analysis

The MusaBAG protein sequences, obtained in the previous step, were analyzed for domains using InterPro online tool (https://www.ebi.ac.uk/interpro/) to annotate the conserved BAG domains^38^. The MusaBAG protein sequences were also aligned with the corresponding AtBAG protein sequences using PROMALS3D multiple sequence and structure alignment server (http://prodata.swmed.edu/promals3d/promals3d.php) to annotate the corresponding domains present in the AtBAGs, such as the IQ motif, KED sequence motif, conserved ExRPGG[ML/VV]QxR motif, as described by Yan et al.^6,39^. The annotated BAG domain of MusaBAG proteins were also confirmed by aligning them with the BAG domain of their respective *Arabidopsis* homologs. The BAG domains of MusaBAGs were aligned to examine their secondary structure-based conservation of amino acid residues along the entire domain, using PROMALS3D multiple sequence and structure alignment server (http://prodata.swmed.edu/promals3d/promals3d.php)^39^.

### Structural analysis

The structures of MusaBAG proteins were obtained by submitting the sequences to Iterative Threading ASSEmbly Refinement (I-TASSER) server^40^. This server predicts the protein structure in a hierarchical method by iterative template-based fragment assembly simulation, using the structural templates provided by Protein Data Bank (PDB). Considering the confidence score (C-score) of the predicted models, the server provides five simulated structures for each of the MusaBAG protein sequences provided. The structure with the highest C-score value was selected, for each of the MusaBAG proteins, for further analysis.

### Phylogenetic analysis and classification

All the 12 MusaBAG proteins of banana were clustered to make a phylogenetic tree using the neighbor joining method of ‘amino acid substitution’ type using Jones–Taylor–Thornton (JTT) model with 1000 bootstrap replications, uniform rating among sites and pairwise deletion gap treatment in MEGA version X^41^. Similarly, all the 28 BAG protein sequences (12 BAG proteins of banana, 8 BAG proteins of *Arabidopsis*, 6 BAG proteins from rice and two mammalian BAG proteins) were analyzed for their phylogenetic relationship by keeping the parameters same as the previous analysis and using Jones-Taylor-Thornton (JTT) model in MEGA version X^41^. As two out-groups, the full-length protein sequence of BAG1 of mouse (MmBAG1) (Uniprot ID—Q60739) and BAG5 (HsBAG5) (Uniprot ID—Q9UL15) of human were retrieved from Uniprot database^42^.

### Prediction of subcellular localization

The subcellular localization of MusaBAG proteins was determined using web-based tools. These include: DeepLoc 1.0 (http://www.cbs.dtu.dk/services/DeepLoc/index.php) that predicts the subcellular localization of eukaryotic proteins using Neural Networks algorithm based on the sequence information^43^. Psort (http://psort1.hgc.jp/form.html) prediction is based on the amino acid sequence and composition^44^. Plant PLoc (http://www.csbio.sjtu.edu.cn/bioinf/plant-multi/) predicts localization based on the gene ontology information, functional domain information, and sequential evolutionary information^45^ whereas YLoc (https://abi-services.informatik.uni-tuebingen.de/yloc/webloc.cgi) is an interpretable server for predicting subcellular localization of proteins^46^. Another software called TargetP 2.0 (http://www.cbs.dtu.dk/services/TargetP-2.0/cite.php) predicts the presence of N-terminal pre-sequences such as signal peptide, mitochondrial transit peptide, chloroplast transit peptide or thylakoid luminal transit peptide but does not provide information on the nuclear or cytoplasm targeting and represents it as other category and provides its scores on the likelihood^47^. WoLF PSORT (https://wolfpsort.hgc.jp/) predicts subcellular localization sites of proteins based on their amino acid sequences and the results are based on the nearest neighbors^48^. The full-length protein sequences of MusaBAG were deposited in these servers and queried for their subcellular localization.

### Cis-element in promoter sequences

Two kilobases region upstream of the transcription start site of each *MusaBAG* coding sequences except for *MusaBAG8* (upto 300 bases) was extracted from the whole genome short-gun sequence database of NCBI and used for the analysis of *cis-*regulatory elements. The *cis*-regulatory elements within these sequences were determined using online tools such as PlantPAN 3.0 (http://plantpan.itps.ncku.edu.tw/) and Plant CARE (http://bioinformatics.psb.ugent.be/webtools/plantcare/html/).

### Digital expression patterns and ESTs

The RNAseq data for each of the *MusaBAGs* is available in the banana genome hub (https://banana-genome-hub.southgreen.fr/)^34^. This data accounts for mRNA expression levels under biotic stress condition i.e., *Fusarium oxysporum* f. sp. *cubense* TR4 (tropical race 4, now *Fusarium odoratissimum*) infected condition in *Musa acuminata* DH Pahang variety (AA), and an abiotic stress condition i.e., osmotic stress in Cavendish Grand Naine variety (AAA), subject to availability of RNA sequencing data in the banana genome hub database. For both biotic and abiotic stress conditions, the average maximum RNA level data was collected for the *MusaBAG* genes and compared with the non-infected or control RNA levels, by plotting them in a grouped bar graph. The significance of difference between non-infected/control versus the test condition (biotic or abiotic stress) was estimated using non parametric t-test (Wilcoxon matched-pairs signed rank test) and the significance of difference between individual *MusaBAG* gene expression under control and test conditions was evaluated using two-way ANOVA (Sidak’s multiple comparisons test). The plotting of graphs and testing of hypotheses for significance of differences was done using GraphPad Prism version 8.0.2 (GraphPad Software, San Diego, California USA; www.graphpad.com).

### Quantitative real time RT-PCR analysis

Banana cv. Rasthali (Silk, AAB group; susceptible to both *Fusarium oxysporum* f. sp. *cubense* Foc race 1 and tropical race 4) were micro-propagated under tissue culture conditions and rooted plants were used in this experiment^49–51^. Rasthali plants were obtained from fields and authenticated by the Karnataka Horticulture Department, Govt. of India. The rooted plants were transferred to fresh MS liquid medium (containing 3% sucrose and pH-5.7) and treated with three different stress conditions so as to observe the differential gene expression pattern of *MusaBAG* genes. For abiotic stress, banana plants were treated with 300 mM sodium chloride or 5% polyethylene glycol (PEG-8000). For biotic stress, each plant was inoculated with 5 × 10^6^ conidia of *Fusarium oxysporum* f. sp. *cubense* race 1 (Foc R1)^52^. Three independent plants were used for each stress treatment. Plants transferred to MS liquid medium without any treatment were used as control. After 72 h of treatment, total RNA was extracted from the root tissue as previously described^13^. The concentration and purity of the extracted total RNA was determined using Infinite M-Plex UV–Vis spectrophotometer (Tecan, Switzerland) and integrity was checked by resolving it on a 2% agarose gel. One microgram of the total RNA was used for first strand cDNA synthesis using ProtoScript^®^ First Strand cDNA Synthesis Kit (New England Biolabs, MA, USA) following manufacturers’ protocol. The cDNA was diluted 1:2.5 and used as a template for qRT-PCR analysis. qRT-PCR was carried out on CFX96 Touch Real-Time PCR Detection System (Bio-Rad Laboratories, USA) using 1 μl of the template DNA and SsoFast EvaGreen Supermix (Bio-Rad Laboratories, USA) following manufacturers’ instructions. Primers specific for *MusaBAG* genes were used alongside *Musa acuminata* elongation factor 1α (*MusaEF1α*) coding sequence for gene expression normalization and subsequent quantification. C_t_ values were retrieved using CFX Manager™ Software. The C_t_ value for each of the *MusaBAG* genes was first normalized with the respective C_t_ value of the *MusaEF1α* gene to obtain ΔC_t_. Then for the entire population under each stress condition, the ΔC_t_ values were normalized against the control population for each of the *MusaBAG* genes to obtain ΔΔC_t_ value and expressed as 2^(−ΔΔCt)^. The significance of the difference between individual *MusaBAG* gene expression under control and stress condition was evaluated using two-way ANOVA (Dunnett’s multiple comparisons test). The plotting of graphs and testing of hypotheses for significance of differences was performed using GraphPad Prism version 8.0.2 (GraphPad Software, San Diego, California USA; www.graphpad.com).

### Protein–protein interactions

The Protein–protein interaction (PPI) networks for MusaBAGs were identified using the STRING search tool (version 11.0) (http://string-db.org) with default parameters. The interactions were derived from sources such as experimentally determined interactions, curated databases, co-expression, text mining and co-occurrence of the proteins^53^.

## Supplementary Information

Supplementary Information.
