# Supplementary material for: Genome-wide in silico characterization and stress induced expression analysis of BcL-2 associated athanogene (BAG) family in Musa spp
Source: Sci Rep. 2022 Jan 12;12:625. doi: 10.1038/s41598-021-04707-5 (PMC8755836; doi:10.1038/s41598-021-04707-5)
Supplement: Supplementary file 1 — Supplementary Information. [file 41598_2021_4707_MOESM1_ESM.pdf]

Supplementary Information S1

In silico analysis of KED domain of MusaBAG7 showing sequence conservation with AtBAG7 and probable calcium and magnesium binding sites.

Structure of KED sequence motif

The KED sequence motif of MusaBAG7 was obtained by a structure based multiple sequence alignment with the KED sequence motif of the corresponding *Arabidopsis* homolog AtBAG7 (Yan et al., 2003). The sequence motif characteristically consists of repeated stretches of charged amino acids such as lysine (K), glutamate (E) and aspartate (D). KED sequence motif of MusaBAG7 is a 200 amino acid stretch sequence which comprises of about 15.6% and 15.5% of lysine residues, 13.1% and 12.4% of glutamate residues and 3% and 5.7% of aspartate residues in MusaBAG7_LOC103973192 and MusaBAG7_LOC103975253 respectively as compared to the AtBAG7 which is 243 amino acid long motif, comprising of 21.4% lysine residues, 16% of glutamate residues and 3.3% of aspartate residues. The alignment KED sequence motif of MusaBAG7 with AtBAG7 shows good alignment (with least gaps and mismatch) towards beginning and end of the motif, however a long stretch of about 35 amino acids, mostly consisting of lysine repeats, was present in AtBAG7, but was absent in both the paralogs of MusaBAG7 (S1 Figure 1).

```
Conservation:        99                  9  9 9   9     9 999  99     9  9   99999 9 9  9
MusaBAG7_3192    1   FGFERSA--I--------ASFKRLRQRAETELCFRDLSDRVASLEFGLGRARSQDVDRKYTWTAEFSGPK   60
MusaBAG7_5253    1   FGFERSA--V--------ASFKRLRERADTELCLRDLSDRVTALELGLGRAHSRDLDRKYTWTAEINGPR   60
AtBAG7           1   --FETVTDLVKIKKSPSSCKYKVIRRRLEPEYPLKYLCDRVSDLESKFDRLVSPKSDRKYTLTKEIKGSG   68
Consensus_aa:        ..FEpsh..l.......tp@K.lRpRh-sEhsh+.LtDRVssLE..hsRh.S.c.DRKYThT.EhpGs.
Consensus_ss:            hhhhhhhh      hhhhhhhhhh hhhhhhhhhhhhhhhhhhhhhh       eeeeehhe

Conservation:           99  9 9      99  9   9  9           99  9    99
MusaBAG7_3192   61   GLGLDRKHRWTATAKAGGERGVKCAVEIKGVEEEEEGFDRKFVWATEAKGVGK----------------  114
MusaBAG7_5253   61   ELGLDRKYKWMAAAKAGGERALKCTAEFKGA--EEDEGFDRKFVWAANGKGVGE----------------  112
AtBAG7          69   ----ERKYKWEAEIQGPLERKYKLEAEIEGSG-------ERKYRWTTEIKGGKKDEEGLKLAALKKEKAK  127
Consensus_aa:        ....-RK@+W.A.hpts.ER.hKh.hEhcGs........-RK@.Whhp.KGs.c................
Consensus_ss:            eeeeehhhe  hhheeeeeeee    hhhh    eeeeeeee                hhhhhhhhhh

Conservation:                 9           99 9 9         9  9             99    9   9  99
MusaBAG7_3192  115   -------RYEK-----------WTAEFKGKGKFSPLSRTYTWAASAKPREEEEGQEKAAVNKEKKKKGKK  166
MusaBAG7_5253  113   -------RNVK-----------WTAEFKGKGKDSPLSRAYTWASSTKPREDDE-EEKAA--KKEKKKEKK  161
AtBAG7         128   AKAIAAAEAEKKKNKNKKKSYNWTTEVKSERENGEVSHTYIIKATTGGEKKKKHEEKEKKEKIETKSKKK  197
Consensus_aa:        .......c..K...........WThEhKtc.c.t.lS+hYhh.toh.sccccc.pEK....KbcpKp.KK
Consensus_ss:        hhhhhhhhhhhhhhhhhh    hhhhh          eeeeeeeee      hhhhhhhhhhhhhhhhhhhhhh

Conservation:         9   9   9 9         99   9999  9        999 99
MusaBAG7_3192  167   EKQGTVHVVEIEVK-----NPGAVAIRKAFTKRCN-----KGKKKE-----  202
MusaBAG7_5253  162   AKEGTVHVVEIEEK-----NPGAIAIRKAFTKRCN-----KGKKKELTPQD  202
AtBAG7         198   EKTRVVVIEEEEEEDDESSEHGAIVLRKAFSRRNGAVRTKKGKNKE-----  243
Consensus_aa:        .Kp.hVhl.EbE.c.....p.GAlhlRKAFo+Rss.....KGKpKE.....
Consensus_ss:        h eeeeeeeeeehhhhhh    hhhhhhhhhh     ee
```

S1 Figure 1 - Multiple sequence alignment of the KED sequence motif of MusaBAG7_3192, MusaBAG7_5253 AtBAG7.

The I-TASSER predicted structures of both the paralogs of MusaBAG7, when analysed for the KED sequence motif, showed presence of multiple α-helices joined by hairpin loops. The electron density structure of this region shows interaction pocket-like structures, suggesting probable interacting with other proteins or nucleotides (S1 Figure 2A and 2B). Previous studies have shown that the *Arabidopsis* homolog of BAG7, interacts with several ER-stress related transcription factors, such as bZIP and WRKY29, followed by nuclear localization (Li et al., 2017). In this structural analyses, we identified two very classical metal ion binding pockets, in both the MusaBAG7s, whose organization in the 3D-space resembles to that of the divalent cation, such as calcium and magnesium binding pocket similar to that of the EF-hand domain of Parvalbumin and few other proteins (Jing et al., 2018). This pocket consists of a specific 3-D arrangement of four aspartate residues and a lysine residue interacting with calcium in parvalbumin (PDB ID – 1B8L) (S1 Figure 2C). In contrast we observed a similar 3-D arrangement of four glutamate (E178, E180, E181, E182) residues and a lysine residue (K175) forming one pocket (S1 Figure 2D) and the other pocket formed by four glutamate (E232, E233, E234, E235) residues and a lysine residue (K229) in MusaBAG7_ LOC103973192 (S1 Figure 2F). In MusaBAG7_ LOC103975253, these corresponding pockets are formed by Glu179, Glu180, Asp181, Glu182 with Lys176 (S1 Figure 2E) and Glu231, Asp232, Asp233, Glu234 with Lys228 (S1 Figure 2G).

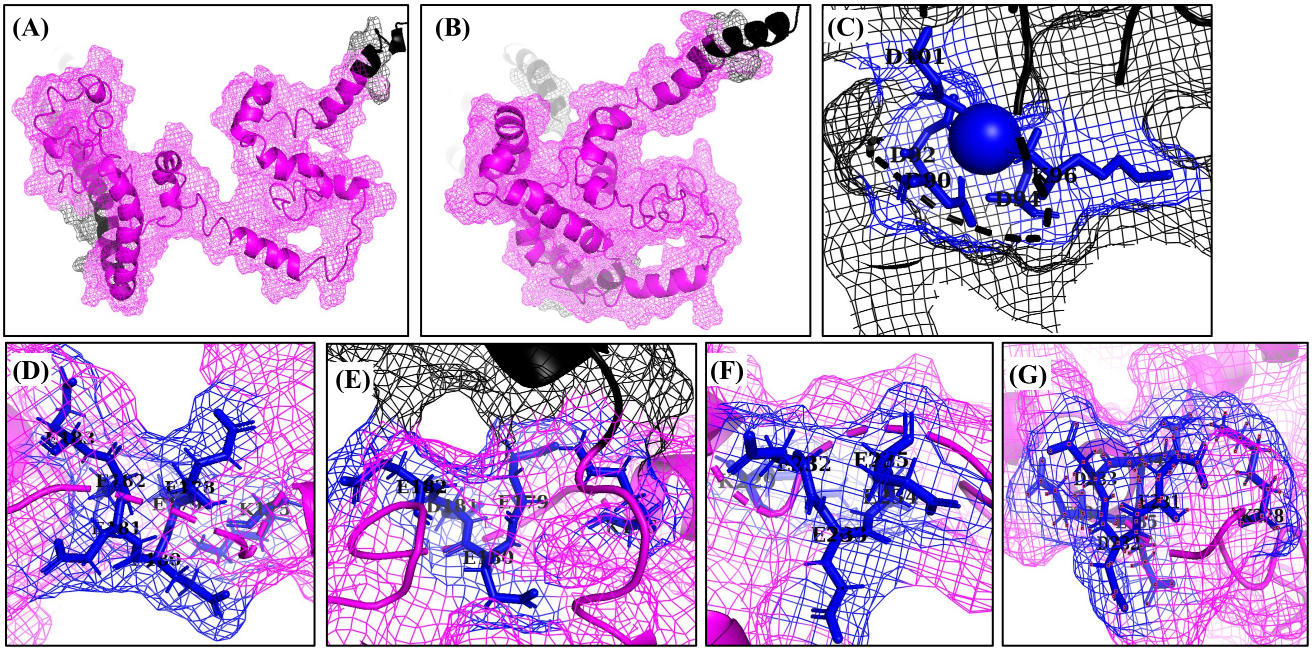

S1 Figure 2 - Structural Analysis of KED sequence motif of MusaBAG7_LOC103973192 (denoted as MusaBAG7_3192) and MusaBAG7_103975253 (denoted as MusaBAG7_5253). (A-B) The electron density structure of the KED sequence motif of MusaBAG7_3192 and MusaBAG_5253 respectively. (C) Typical residues (highlighted in blue) of the EF hand domain of parvalbumin interacting with calcium ion (blue sphere). (D and F) A pocket consisting of similar organization of residues as found in EF hand domain of parvalbumin, in the structure of MusaBAG7_3192 and (E and G) – MusaBAG7_5253.

Metal Ion Docking analysis

Following the identification of possible metal ion binding sites, we checked of presence of calcium and magnesium ion docking sites within the KED sequence motif of MusaBAG7, using MIB: Metal ion binding and docking server. This server predicted about three probable calcium binding sites and four probable magnesium binding sites within the KED motif region for MusaBAG7_ LOC103973192 (S1 Table 1). In corresponding MusaBAG7_ LOC103975253 paralog, there were fourteen probable calcium binding sites and seven probable magnesium binding sites were predicted (S1 Table 1). Out of these predicted, calcium and magnesium binding sites, one each out of the three calcium binding sites in MusaBAG7_ LOC103973192 KED region and out of the four calcium binding sites in MusaBAG7_ LOC103973192 KED region were predicted to be those pockets, we identified (discussed in the previous section). Similarly, six out of the fourteen magnesium binding sites in MusaBAG7_ LOC103973192 KED region and four out the seven magnesium binding sites in MusaBAG7_ LOC103973192 KED region were predicted to be those pockets, we identified.

The molecular docking of the predicted calcium and magnesium binding for both the paralogs of MusaBAG7, were shown for reference (S1 Figure 3 and S1 Figure 4)

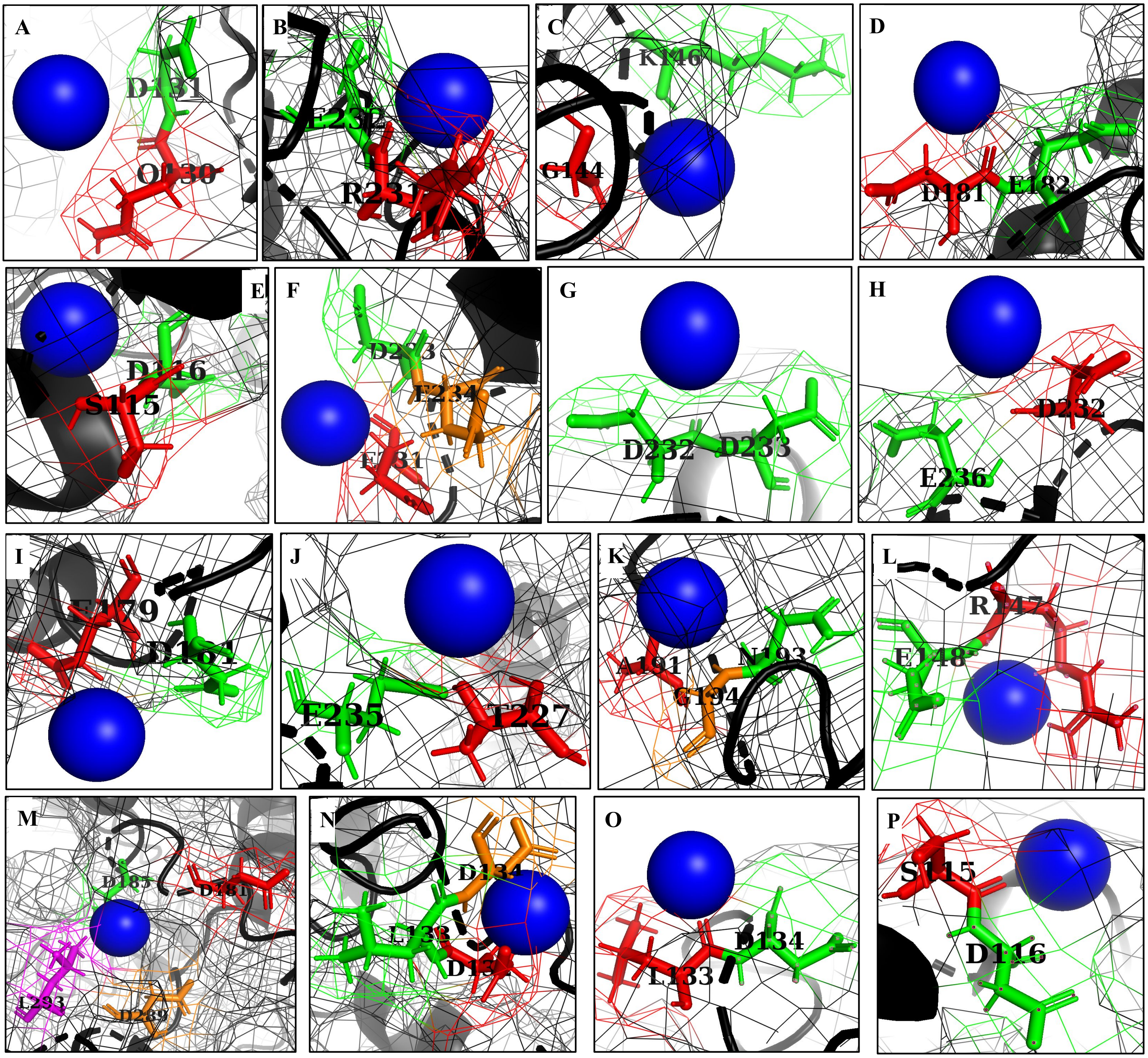

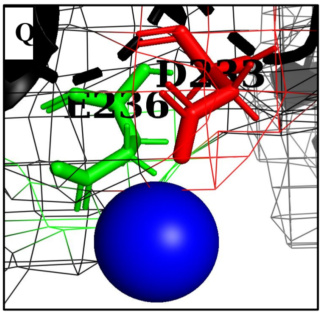

S1 Figure 3 – Molecular docking structures of calcium ion (blue spheres) at probable interacting sites within the KED sequence motif of MusaBAG7, based on prediction, using MIB: Metal Ion-Binding Site Prediction and Docking Server (details in S2 Table 1). The interacting residues are labelled in the structure and highlighted in green (Ist residue), red (IInd residue), orange (IIIrd residue, if any) and magenta (IVth residue, if any). (A-C) – Calcium docked structures in the KED domain of MusaBAG7_LOC103973192. (D-Q) – Calcium docked structures in the KED domain of MusaBAG7_LOC103975253.

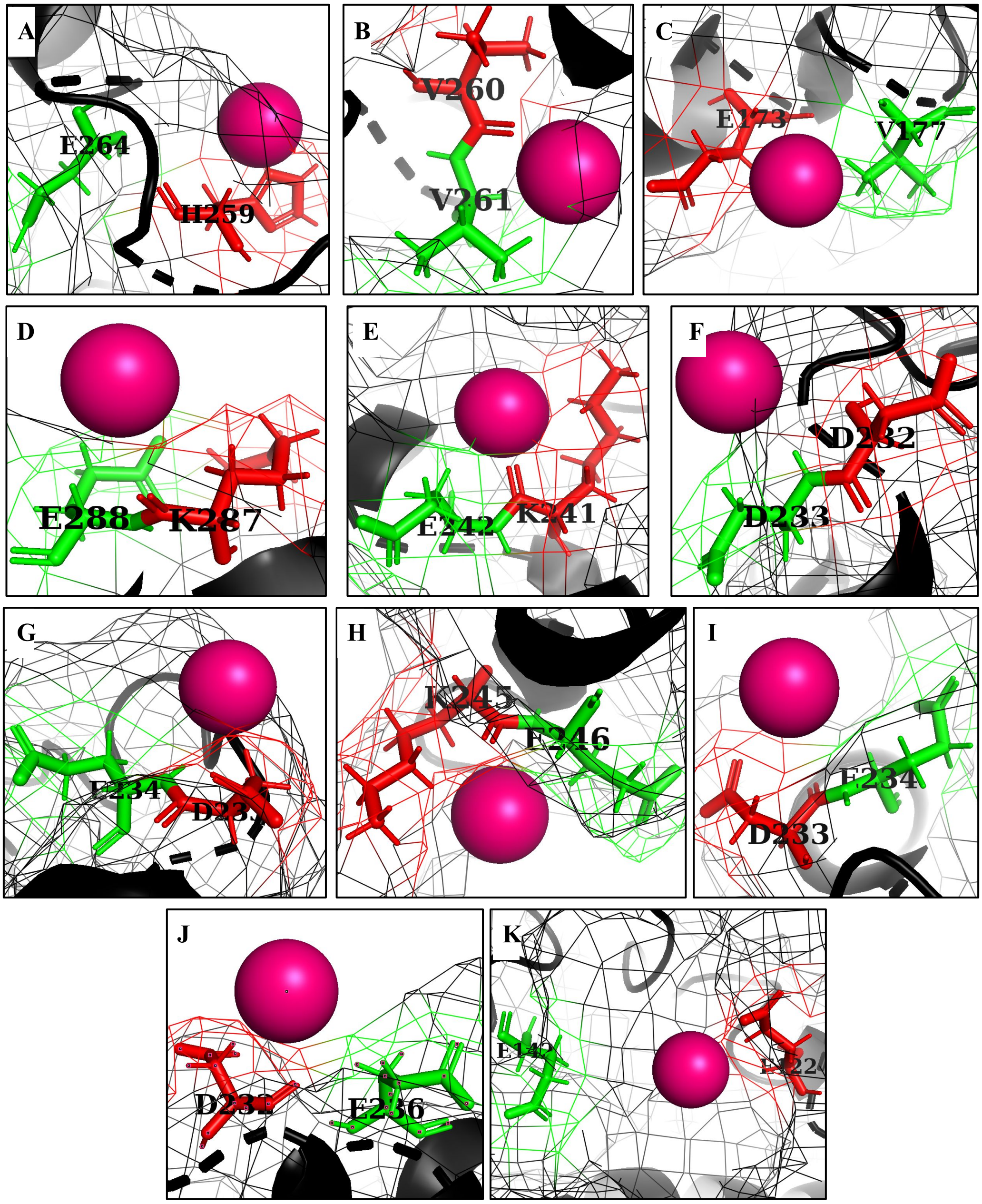

S1 Figure 4 – Molecular docking structures of magnesium ion (pink spheres) at probable interacting sites within the KED sequence motif of MusaBAG7, based on prediction, using MIB: Metal Ion-Binding Site Prediction and Docking Server (details in S2 Table 2). The interacting residues are labelled in the structure and highlighted in green (Ist residue), red (IInd residue) and magenta (IVth residue, if any). (A-D) – Magnesium docked structures in the KED domain of MusaBAG7_LOC103973192. (E-K) – Magnesium docked structures in the KED domain of MusaBAG7_LOC103975253.

Calcium Ion Binding site				
**For MusaBAG7_LOC103973192**				
**Sl. No.**	**Binding residues**	**Template**	**Score**	**Template PDB Id**
1	130Q , 131D	Alkaline Phosphatase from *P. aeruginosa*	1.33	1AKL_4
2	231R , 232E	ATP bound Nitrogenase protein complex	1.225	1G21_B0
3	144G , 146K	Porin from *R. capsulatus*	1.181	2POR_2
**For MusaBAG7_LOC103975253**				
1	181D , 182E	Extracellular domain of the LDL receptor	1.733	1N7D_A3
2	115S , 116D	Carboxypeptidase T	1.697	1OBR_1
3	231E , 233D , 234E	Family 9 Carbohydrate binding module of Xylanase	1.496	1I82_A0
4	232D , 233D	Endoglucanase D	1.477	1CLC_1
5	232D , 236E	Endoglucanase CEL5A	1.451	1E5J_A2
6	181D , 182E	Carboxypeptidase T	1.399	1OBR_0
7	179E , 181D	Metalloprotease	1.313	1DTH_A0
8	227T , 235E	Annexin III	1.3	1AXN_1
9	191A , 193N , 194G	Iota-Carrageenase	1.29	1KTW_A2
10	147R , 148E	ATP bound Nitrogenase protien complex	1.288	1G21_B0
11	179E , 185D , 289D , 293L	S-S-lambda-shaped TRANS and CIS interactions of cadherins	1.287	1Q5C_CA
12	132D , 133L , 134D	Quinoenzyme: Copper amine oxidase	1.276	1OAC_B0
13	133L , 134D	C2 domain from protein Kinase C	1.227	1DSY_A0
14	115S , 116D	Intein Homing Endonuclease PI-Scel	1.211	1LWS_A1
15	233D , 236E	Human H Chain ferritin	1.21	2FHA_1
**Magnesium Ion Binding site**				
**For MusaBAG7_LOC103973192**				
1	259H , 264E	dTDP-Manganese complex of SPSA	1.438	1H7Q_A0
2	260V , 261V	mammalian 20S proteasome	1.426	1IRU_G2
3	173E , 177V	mammalian 20S proteasome	1.347	1IRU_G2
4	287K , 288E	Nitrogenase MO-FE protein	1.185	1H1L_B1
**For MusaBAG7_LOC103975253**				
1	241K , 242E	Nitrogenase MO-FE protein	1.507	1H1L_B1
2	232D , 233D	Glutamine Phosphoribosylpyrophosphate amidotransferase	1.466	1ECB_C0
3	233D , 234E	RUBISCO	1.368	1BWV_A0
4	245K , 246E	Nitrogenase MO-FE protein	1.359	1H1L_B1
5	233D , 234E	Anthranilate phosphoribosyl-transferase	1.317	1GXB_D0
6	232D , 236E	Acetohydroxy acid isomereductase	1.227	1YVE_I0
7	122E , 142E	ADP ribose-pyrophosphatase	1.18	1KHZ_B1

S1 Table 1 – Calcium and magnesium ion binding prediction and docking within the KED sequence motif of MusaBAG7_LOC103973192 and MusaBAG7_LOC103975253, done by MIB: Metal Ion-Binding Site Prediction and Docking Server, based on fragment transformation method. The interacting residues (denoted as binding residues), template used for predicting the particular interaction, along with their PDB information and score of the interaction are mentioned.

Materials and Method

Identification of KED sequence motif in MusaBAG7

The presence of KED sequence motif was characterized at the protein sequence level in the *Arabidopsis* homolog of BAG7, namely AtBAG7 by Yan et al. (2003). This sequence was aligned with both the protein sequence of MusaBAG7 paralogs, using structure based multiple sequence alignment, using PROMALS3D multiple sequence and structure alignment server (*http://prodata.swmed.edu/promals3d/promals3d.php*) (Pei et al*.,* 2008), to identify the KED sequence motif in MusaBAG7. The amino acid residue composition for the KED sequence of AtBAG7 and MusaBAG7 paralogs was analysed using Protein Identification and Analysis Tools on the ExPASy Server (Gasteiger et al., 2005).

Structural analysis of MusaBAG7 KED sequence motif

The structures of the MusaBAG7 paralogs were obtained by submitting the sequences to Iterative Threading ASSEmbly Refinement (I-TASSER) server. Considering the confidence score (C- score) of the predicted models, the server provides five simulated structures for each of the MusaBAG protein sequences provided. The structure with the highest C-score value was selected, for each of the MusaBAG7 proteins, for further analysis. The structure of EF hand domain of Parvabumin was obtained from protein data bank (Id – 1B8L). The structural representation including highlighting specific residues, was done using the PyMol molecular graphics sytem v2.4 (Schrödinger L, DeLano W. PyMOL [Internet]. 2020. Available from: http://www.pymol.org/pymol).

Metal ion binding prediction and docking

The metal ion binding site prediction and molecular docking for MusaBAG7 was done by submitting the PDB file obtained from I-TASSER server to the MIB: Metal Ion-Binding Site Prediction and Docking Server (Lin et al., 2016). This server predicts metal ion binding residues and build metal ion docked 3D structures, by comparing with the pre-constructed metal ion binding templates, based on protein data bank structures. The predicted calcium and magnesium ion binding sites within the KED sequence motif were selected for analysis. The provided metal ion docked structures were represented by highlighting the particular predicted binding residues using the PyMol molecular graphics sytem v2.4 (Schrödinger L, DeLano W. PyMOL [Internet]. 2020. Available from: http://www.pymol.org/pymol).

References

1. Li Y, Williams B, Dickman M. Arabidopsis B‐cell lymphoma2 (Bcl‐2)‐associated athanogene 7 (BAG 7)‐mediated heat tolerance requires translocation, sumoylation and binding to WRKY 29. New Phytologist. 2017, 214(2):695-705.
2. Hara K, Yagi M, Koizumi N, Kusano T, Sano H. Screening of wound-responsive genes identifies an immediate early expressed gene encoding a highly charged protein in mechanically wounded tobacco plants. Plant and Cell Physiology. 2000. 41(6):684-91.
3. Yan J, He C, Zhang H. The BAG-family proteins in Arabidopsis thaliana. Plant Science. 2003 Jul 1;165(1):1-7.
4. Jing Z, Liu C, Qi R, Ren P. Many-body effect determines the selectivity for Ca2+ and Mg2+ in proteins. Proceedings of the National Academy of Sciences. 2018. 115(32):E7495-501.
5. Pei J, Kim BH, Grishin NV. PROMALS3D: a tool for multiple protein sequence and structure alignments. Nucleic acids research. 2008 Apr 1;36(7):2295-300.
6. Gasteiger E, Hoogland C, Gattiker A, Wilkins MR, Appel RD, Bairoch A. Protein identification and analysis tools on the ExPASy server. The proteomics protocols handbook. 2005:571-607.
7. Schrödinger L, DeLano W. PyMOL [Internet]. 2020. Available from: http://www.pymol.org/pymol

Supplementary Figure S2

Supplementary Figure S2: Protein-protein interaction network of individual MusaBAG proteins. All MusaBAG1 paralogs and MusaBAG2 have similar protein interactors (A). The protein interactors of MusaBAG4 (B), MusaBAG5 (C) and MusaBAG6 (D) are represented using STRING analysis at medium confidence levels. MusaBAG7 (E) and MusaBAG8 (F) interactors are also predicted at medium confidence levels.

(A)

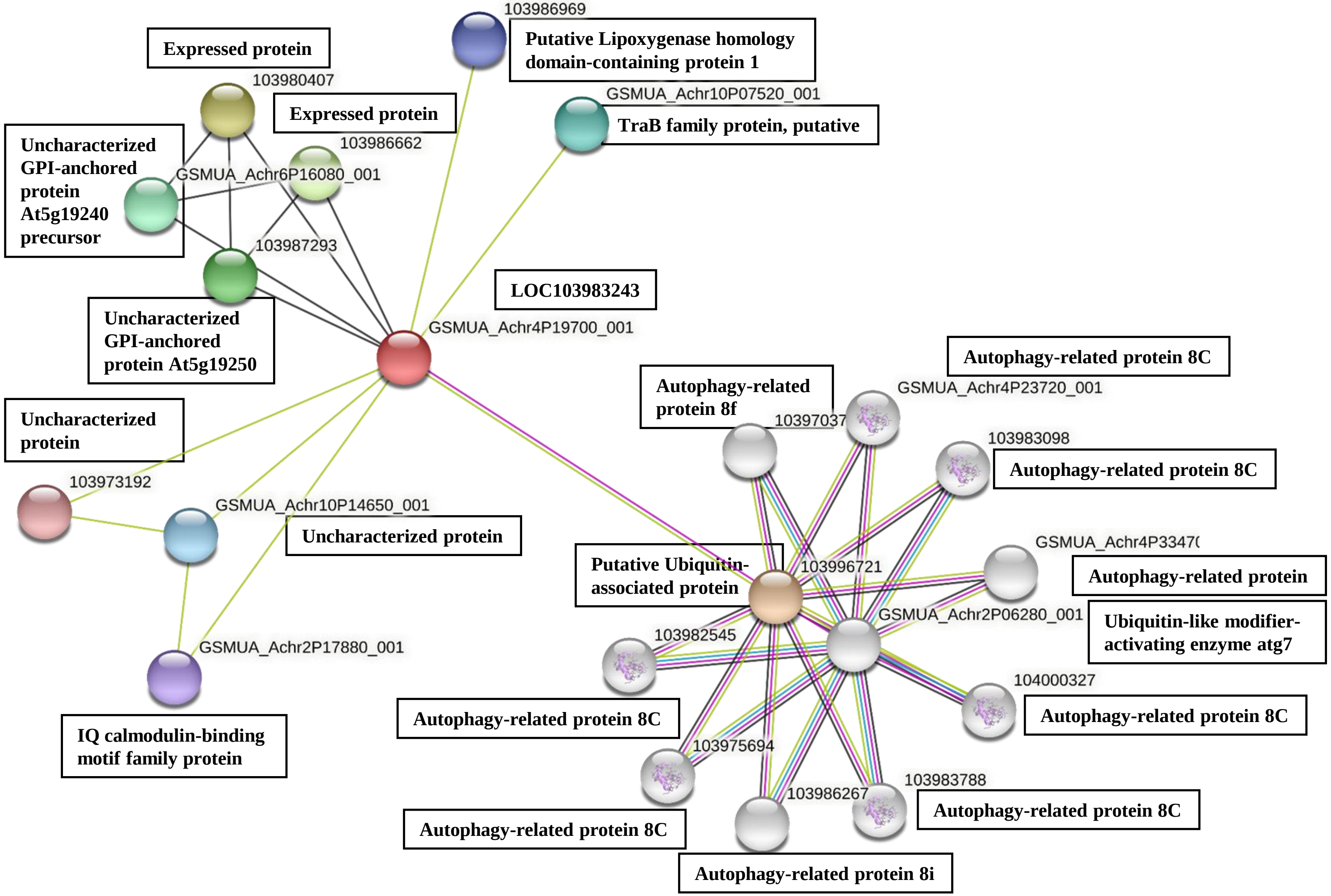

Expressed protein
103980407
Expressed protein
103986662
GSMUA_Achr6P16080_001
Uncharacterized GPI-anchored protein At5g19240 precursor
103987293
Uncharacterized GPI-anchored protein At5g19250
103986969
Putative Lipoxygenase homology domain-containing protein 1
GSMUA_Achr10P07520_001
TraB family protein, putative
LOC103983243
GSMUA_Achr4P19700_001
Uncharacterized protein
103973192
GSMUA_Achr10P14650_001
Uncharacterized protein
GSMUA_Achr2P17880_001
IQ calmodulin-binding motif family protein
Autophagy-related protein 8C
GSMUA_Achr4P23720_001
Autophagy-related protein 8f
10397037
103983098
Autophagy-related protein 8C
GSMUA_Achr4P3347(
Autophagy-related protein
Putative Ubiquitin-associated protein
103996721
GSMUA_Achr2P06280_001
Ubiquitin-like modifier-activating enzyme atg7
103982545
104000327
Autophagy-related protein 8C
Autophagy-related protein 8C
103975694
103986267
103983788
Autophagy-related protein 8C
Autophagy-related protein 8C
Autophagy-related protein 8i

MusaBAG1, 2

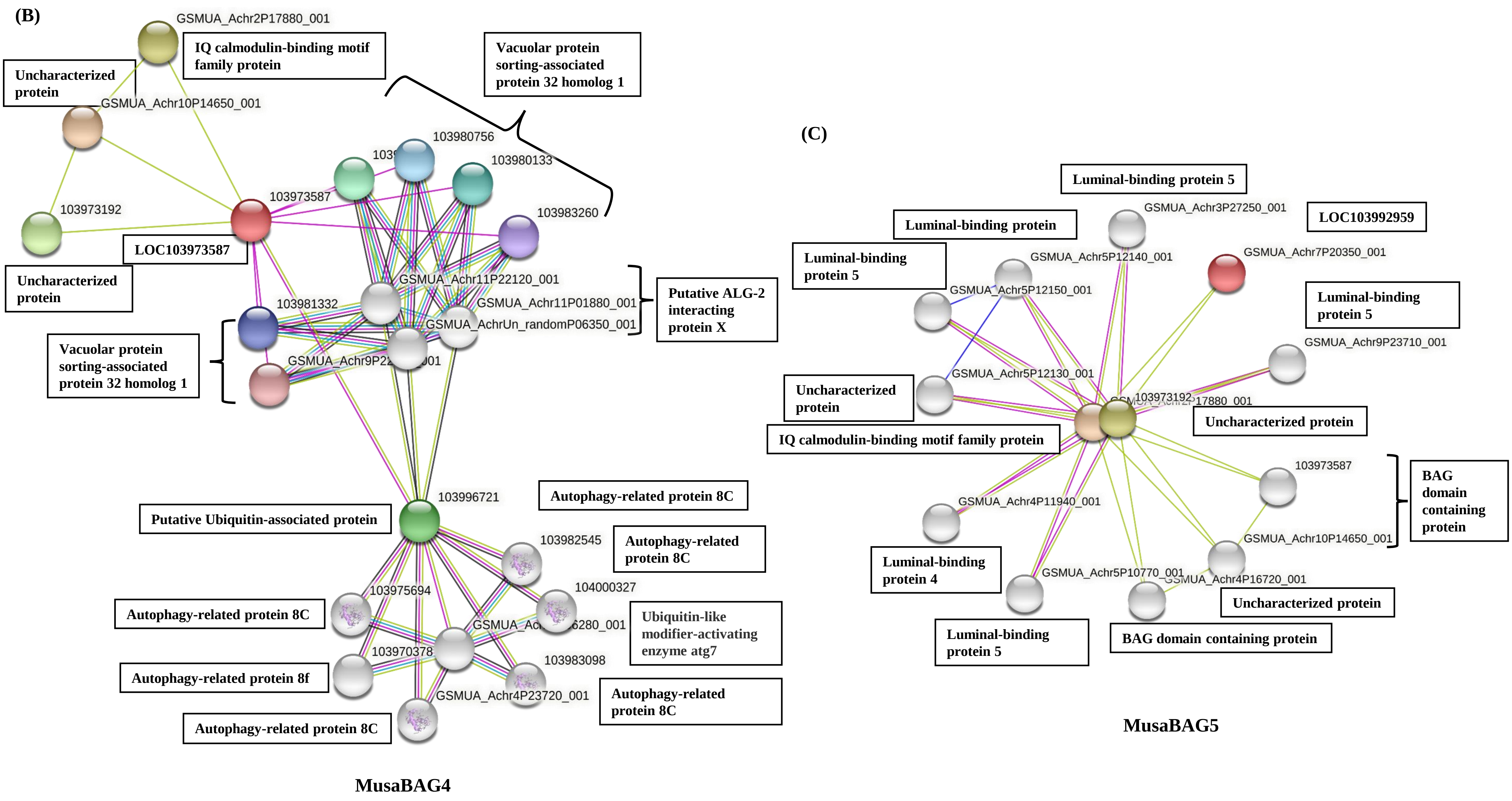
(B)
GSMUA_Achr2P17880_001
IQ calmodulin-binding motif family protein
Uncharacterized protein
GSMUA_Achr10P14650_001
Vacuolar protein sorting-associated protein 32 homolog 1
103980756
103980133
103973587
103983260
103973192
LOC103973587
Uncharacterized protein
GSMUA_Achr11P22120_001
103981332
GSMUA_Achr11P01880_001
GSMUA_AchrUn_randomP06350_001
Putative ALG-2 interacting protein X
Vacuolar protein sorting-associated protein 32 homolog 1
103996721
Autophagy-related protein 8C
Putative Ubiquitin-associated protein
103982545
Autophagy-related protein 8C
104000327
103975694
Autophagy-related protein 8C
Ubiquitin-like modifier-activating enzyme atg7
103970378
103983098
Autophagy-related protein 8f
GSMUA_Achr4P23720_001
Autophagy-related protein 8C
Autophagy-related protein 8C
MusaBAG4
(C)
Luminal-binding protein 5
GSMUA_Achr3P27250_001
LOC103992959
Luminal-binding protein
GSMUA_Achr7P20350_001
Luminal-binding protein 5
GSMUA_Achr5P12140_001
GSMUA_Achr5P12150_001
Luminal-binding protein 5
GSMUA_Achr9P23710_001
GSMUA_Achr5P12130_001
Uncharacterized protein
103973192
Uncharacterized protein
IQ calmodulin-binding motif family protein
103973587
BAG domain containing protein
GSMUA_Achr4P11940_001
GSMUA_Achr10P14650_001
Luminal-binding protein 4
GSMUA_Achr5P10770_001
GSMUA_Achr4P16720_001
Uncharacterized protein
Luminal-binding protein 5
BAG domain containing protein
MusaBAG5

(D)

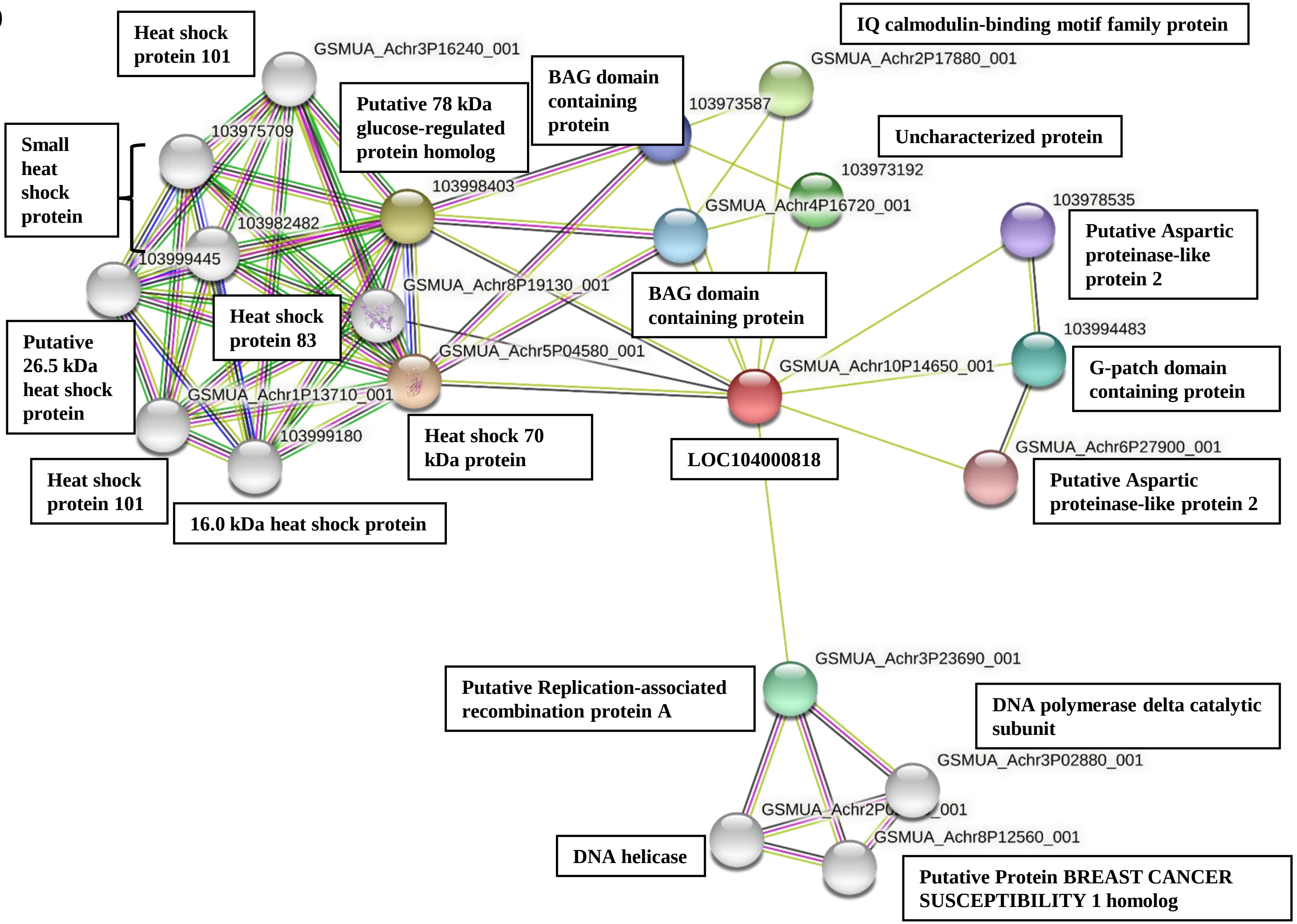
Heat shock protein 101
GSMUA_Achr3P16240_001
Small heat shock protein
103975709
Putative 78 kDa glucose-regulated protein homolog
BAG domain containing protein
103973587
IQ calmodulin-binding motif family protein
GSMUA_Achr2P17880_001
Uncharacterized protein
103973192
103998403
103982482
103999445
GSMUA_Achr4P16720_001
103978535
Putative Aspartic proteinase-like protein 2
GSMUA_Achr8P19130_001
BAG domain containing protein
Heat shock protein 83
Putative 26.5 kDa heat shock protein
GSMUA_Achr5P04580_001
GSMUA_Achr10P14650_001
103994483
G-patch domain containing protein
GSMUA_Achr1P13710_001
103999180
Heat shock 70 kDa protein
LOC104000818
GSMUA_Achr6P27900_001
Putative Aspartic proteinase-like protein 2
Heat shock protein 101
16.0 kDa heat shock protein
GSMUA_Achr3P23690_001
Putative Replication-associated recombination protein A
DNA polymerase delta catalytic subunit
GSMUA_Achr3P02880_001
GSMUA_Achr8P12560_001
DNA helicase
Putative Protein BREAST CANCER SUSCEPTIBILITY 1 homolog

MusaBAG6

(E)

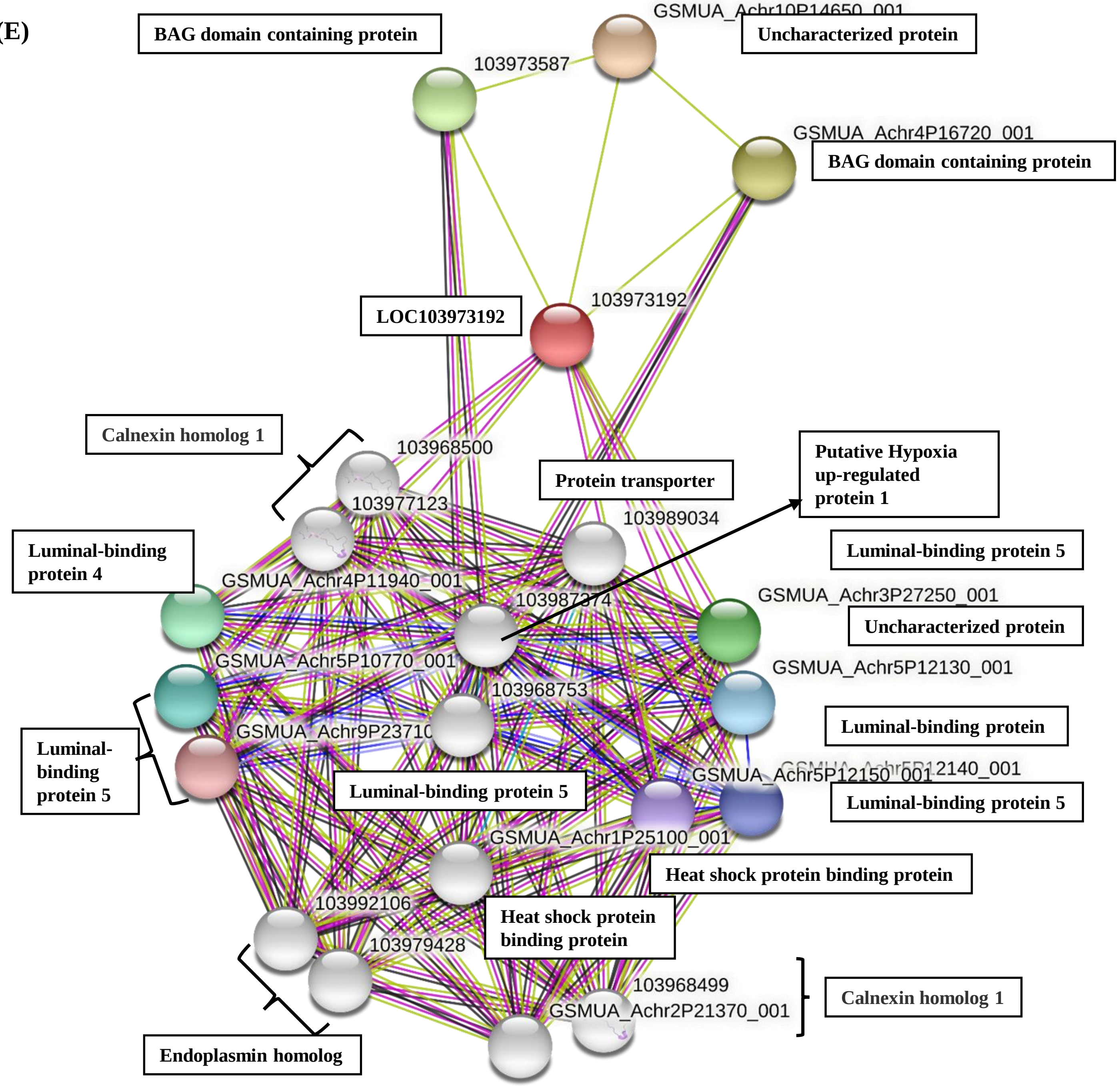
BAG domain containing protein
103973587
GSMUA_Achr10P14650_001
Uncharacterized protein
GSMUA_Achr4P16720_001
BAG domain containing protein
LOC103973192
103973192
Calnexin homolog 1
103968500
103977123
Protein transporter
Putative Hypoxia up-regulated protein 1
103989034
Luminal-binding protein 4
GSMUA_Achr4P11940_001
Luminal-binding protein 5
103987374
GSMUA_Achr3P27250_001
Uncharacterized protein
GSMUA_Achr5P10770_001
GSMUA_Achr5P12130_001
103968753
Luminal-binding protein
GSMUA_Achr9P23710
Luminal-binding protein 5
Luminal-binding protein 5
GSMUA_Achr5P12150_001
GSMUA_Achr5P12140_001
Luminal-binding protein 5
GSMUA_Achr1P25100_001
Heat shock protein binding protein
103992106
Heat shock protein binding protein
103979428
103968499
GSMUA_Achr2P21370_001
Calnexin homolog 1
Endoplasmin homolog

MusaBAG7

(F)

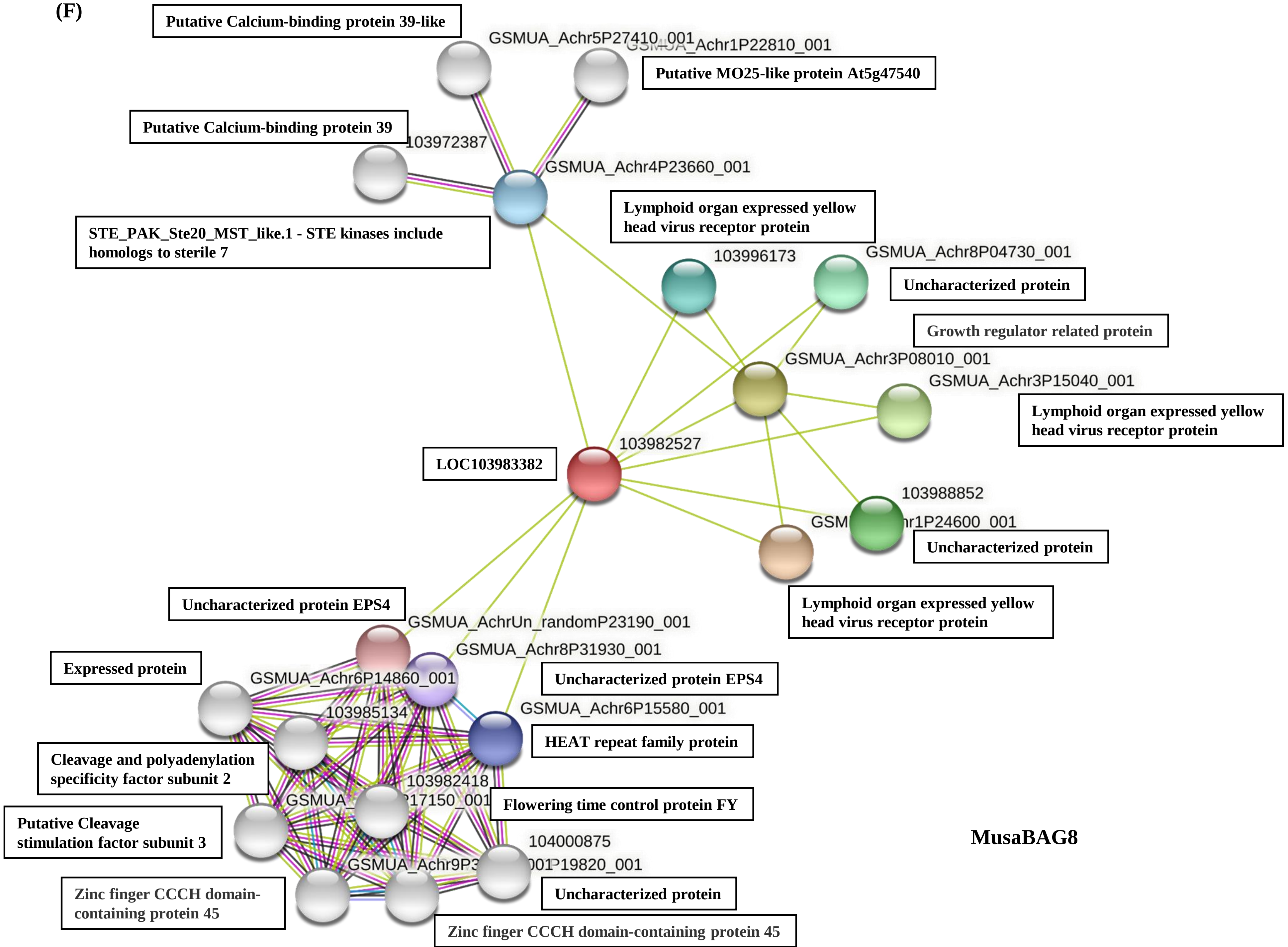
Putative Calcium-binding protein 39-like
GSMUA_Achr5P27410_001
GSMUA_Achr1P22810_001
Putative MO25-like protein At5g47540
Putative Calcium-binding protein 39
103972387
GSMUA_Achr4P23660_001
STE_PAK_Ste20_MST_like.1 - STE kinases include homologs to sterile 7
Lymphoid organ expressed yellow head virus receptor protein
103996173
GSMUA_Achr8P04730_001
Uncharacterized protein
Growth regulator related protein
GSMUA_Achr3P08010_001
GSMUA_Achr3P15040_001
Lymphoid organ expressed yellow head virus receptor protein
103982527
LOC103983382
103988852
Uncharacterized protein
Lymphoid organ expressed yellow head virus receptor protein
Uncharacterized protein EPS4
GSMUA_AchrUn_randomP23190_001
GSMUA_Achr8P31930_001
Uncharacterized protein EPS4
Expressed protein
GSMUA_Achr6P14860_001
103985134
GSMUA_Achr6P15580_001
HEAT repeat family protein
Cleavage and polyadenylation specificity factor subunit 2
103982418
Flowering time control protein FY
Putative Cleavage stimulation factor subunit 3
104000875
Uncharacterized protein
Zinc finger CCCH domain-containing protein 45
Zinc finger CCCH domain-containing protein 45

MusaBAG8
